# Imipenem heteroresistance but not tolerance in *Haemophilus influenzae* during chronic lung infection associated with chronic obstructive pulmonary disease

**DOI:** 10.3389/fmicb.2023.1253623

**Published:** 2023-12-20

**Authors:** Celia Gil-Campillo, Aida González-Díaz, Beatriz Rapún-Araiz, Oihane Iriarte-Elizaintzin, Iris Elizalde-Gutiérrez, Ariadna Fernández-Calvet, María Lázaro-Díez, Sara Martí, Junkal Garmendia

**Affiliations:** ^1^Instituto de Agrobiotecnología, Consejo Superior de Investigaciones Científicas (IdAB-CSIC)-Gobierno de Navarra, Mutilva, Spain; ^2^Centro de Investigación Biomédica en Red de Enfermedades Respiratorias (CIBERES), Madrid, Spain; ^3^Conexion Nanomedicina CSIC (NanomedCSIC), Madrid, Spain; ^4^Microbiology Department, Hospital Universitari Bellvitge, IDIBELL-UB, L’Hospitalet de Llobregat, Barcelona, Spain

**Keywords:** *Haemophilus influenzae*, antibiotic resistance, antibiotic heteroresistance, antibiotic tolerance, imipenem, therapeutic failure

## Abstract

Antibiotic resistance is a major Public Health challenge worldwide. Mechanisms other than resistance are described as contributors to therapeutic failure. These include heteroresistance and tolerance, which escape the standardized procedures used for antibiotic treatment decision-making as they do not involve changes in minimal inhibitory concentration (MIC). *Haemophilus influenzae* causes chronic respiratory infection and is associated with exacerbations suffered by chronic obstructive pulmonary disease (COPD) patients. Although resistance to imipenem is rare in this bacterial species, heteroresistance has been reported, and antibiotic tolerance cannot be excluded. Moreover, development of antibiotic heteroresistance or tolerance during within-host *H. influenzae* pathoadaptive evolution is currently unknown. In this study, we assessed imipenem resistance, heteroresistance and tolerance in a previously sequenced longitudinal collection of *H. influenzae* COPD respiratory isolates. The use of Etest, disc diffusion, population analysis profiling, tolerance disc (TD)-test methods, and susceptibility breakpoint criteria when available, showed a significant proportion of imipenem heteroresistance with differences in terms of degree among strains, absence of imipenem tolerance, and no specific trends among serial and clonally related strains could be established. Analysis of allelic variation in the *ftsI*, *acrA*, *acrB*, and *acrR* genes rendered a panel of polymorphisms only found in heteroresistant strains, but gene expression and genome-wide analyses did not show clear genetic traits linked to heteroresistance. In summary, a significant proportion of imipenem heteroresistance was observed among *H. influenzae* strains isolated from COPD respiratory samples over time. These data should be useful for making more accurate clinical recommendations to COPD patients.

## 1 Introduction

Antimicrobial resistance (AMR) is one of the most serious threats to Public Health and health care. Recent studies based on predictive statistical models estimated 4.95 million deaths associated with bacterial AMR in 2019, including 1.27 million deaths attributable to bacterial AMR. Among those, lower respiratory infections accounted for more than 1.5 million deaths associated with resistance in 2019, making it the most burdensome infectious syndrome (Murray et al., 2022). Resistant bacterial cells survive antibiotic treatment by carrying resistance factor(s) that allow growth at high antibiotic concentrations regardless of treatment duration. However, mechanisms other than resistance contribute to therapeutic failure. Heteroresistance leads to a fraction of the bacterial population displaying a substantial increase in minimal inhibitory concentration (MIC) value, meaning that those cells are less susceptible to the antibiotic and can grow in its presence (El-Halfawy and Valvano, 2015; Andersson et al., 2019; Band and Weiss, 2019). Conversely, tolerance is the ability of antibiotic-susceptible bacteria to survive during transient exposure to high concentrations of a bactericidal antibiotic, whereas persistence is the ability of a clonal bacterial subpopulation to survive transient exposure to high concentrations of antibiotic, i.e., tolerance at the subpopulation level (Brauner et al., 2016; Balaban et al., 2019; Dewachter et al., 2019). Such phenomena escape standardized procedures used for antibiotic treatment decision-making as they do not involve MIC changes.

Nontypeable *Haemophilus influenzae* (NTHi) is a pathobiont that commonly resides in the human nasopharynx, from which it can cause otitis media, conjunctivitis, sinusitis and lower respiratory infections in children; exacerbations of chronic obstructive pulmonary disease (COPD) and cystic fibrosis (CF) in adults; and invasive disease in neonates, immunocompromised adults, and the elderly (Duell et al., 2016; Ahearn et al., 2017; Jalalvand and Riesbeck, 2018; Su et al., 2018). This opportunistic microorganism often develops resistance to β-lactam antibiotics by several mechanisms including β-lactamase production and polymorphisms in the penicillin-binding protein 3 (PBP3) (Parr and Bryan, 1984). Cephalosporins have been used since the emergence of ampicillin-resistant strains, and carbapenems are currently used as an alternative to extended-spectrum cephalosporins for initial empiric treatment of severe infections (Cherkaoui et al., 2018a,b). To date, resistance of *H. influenzae* to imipenem remains low (García-Cobos et al., 2014; Lâm et al., 2020; Potts et al., 2022; Safari et al., 2022), and tolerance has not been reported. However, limitations of the broth microdilution method seem to underestimate the extent of imipenem heteroresistance in *H. influenzae*, as previously reported for invasive and respiratory isolates (Cerquetti et al., 2007; Cherkaoui et al., 2017, 2018a,b; Lâm et al., 2020). Although not formally demonstrated, imipenem heteroresistance may be linked to a combination of altered PBP3 and drug fluxes (Cherkaoui et al., 2017). Moreover, antibiotic heteroresistance or tolerance could be pathoadaptive traits of *H. influenzae*, facilitating persistence in patients by decreasing antimicrobial susceptibility. This aspect is currently unknown.

Here, we used a previously sequenced longitudinal set of COPD strains (Moleres et al., 2018) to address open questions about antibiotic heteroresistance and tolerance during chronic *H. influenzae* infection. Imipenem tolerance was not detected among the tested *H. influenzae* respiratory isolates. However, imipenem heteroresistance seems to be a frequent, complex and likely multifactorial phenotype that should be taken into account in the clinical practice.

## 2 Results

### 2.1 *Haemophilus influenzae* imipenem heteroresistance but not tolerance during COPD chronic lung infection

**Figure 1:**
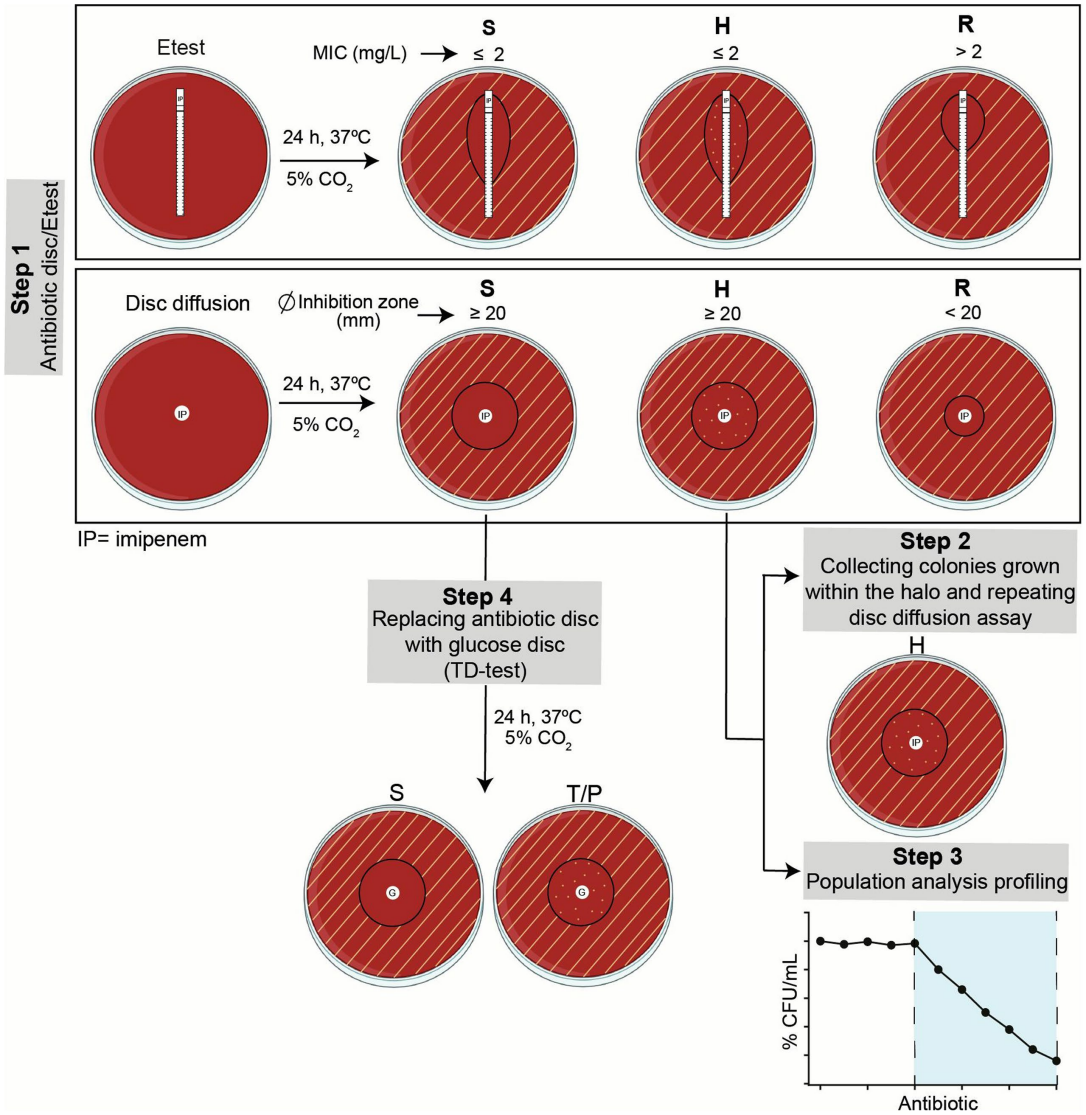
Summary of workflow. Step 1, Etest and disc diffusion assays were run in parallel to screen imipenem susceptibility, resistance or heteroresistance. Growth inhibition areas were read following EUCAST guidelines, as indicated. Heteroresistant strains were further assessed in Steps 2 and 3. Susceptible strains were screened in terms of tolerance by TD-test in Step 4. S, susceptible; H, heteroresistant; R, resistant; T, tolerant; P, persistent.

**Figure 2:**
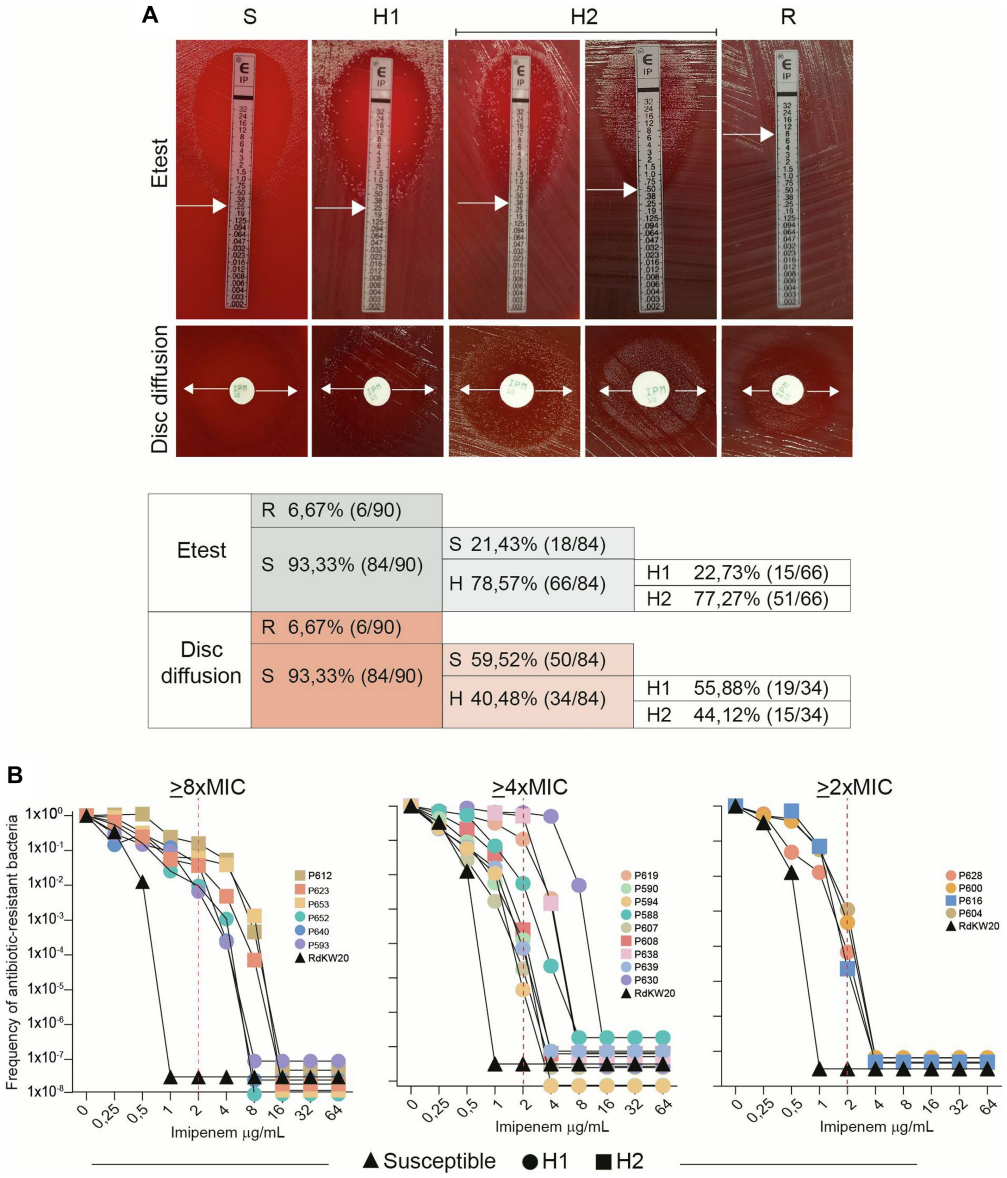
*H. influenzae* imipenem heteroresistance is a heterogeneous phenotype. **(A)** Distribution of susceptibility (S), heteroresistance (H) and resistance (R) in longitudinally isolated NTHi respiratory strains. Etest and disc diffusion methods were used. Heteroresistance was visually heterogeneous among strains, and two categories were established: H1 refers to strains where growth was observed at the edge of the inhibition zone; H2 refers to strains where growth was observed all over the inhibition zone. White arrows indicate growth inhibition used for MIC determination. **(B)** Population analysis profiling (PAP). Nineteen strains shown to be heteroresistant by disc diffusion assay were tested (13 H1 and 6 H2); RdKW20 was used as a susceptible control. Black triangles represent RdKW20 growth; circles are used for H1 strains; squares are used for H2 strains. Data corresponding to one representative experiment are shown. Imipenem clinical breakpoint (2 μg/mL) is indicated with dashed red lines. Strain respective imipenem MIC (μg/mL), according to Etest data: P612, 0.5; P623, 0.38; P653, 0.5; P652, 0.25; P640, 0.5; P593, 0.38; P619, 0.75; P590, 0.38; P594, 0.5; P588, 0.75; P607, 0.5; P608, 0.5; P638, 0.75; P639, 0.5; P630, 1.5; P628, 0.75; P600, 0.75; P616, 1; P604, 1.

Imipenem susceptibility was assessed in a previously sequenced longitudinal set of NTHi respiratory strains (Moleres et al., 2018) by using the Etest and disc diffusion methods (Figure 1 step 1). As expected, most strains (93.3%) were susceptible according to EUCAST guidelines (Figure 2A, Table 1). However, growth of colonies inside the inhibition zone, suggesting the presence of heteroresistant populations, was observed in a significant proportion of susceptible strains, 78.57 and 40.48%, based on Etest and disc diffusion data, respectively. Strains showing heteroresistance by disc diffusion also resulted heteroresistant by Etest. Heteroresistance was heterogeneous among strains, which led us to establish categories upon repeated visual inspection based on the amount and relative location of colonies grown in the inhibition area: H1 refers to strains where colony growth was observed at the edge of the inhibition zone; H2 refers to strains where colony growth was observed, to a higher or lower extent, inside the entire inhibition zone (Figure 2A). Only two strains were β-lactamase positive, P640 and P671, H1 and susceptible, respectively.

**Table 1:** Summary of imipenem susceptibility testing (Etest and disc diffusion), and FtsI, ArcA, ArcB, and ArcR variant distribution across a NTHi longitudinal respiratory strain collection.

			Etest 24 h		Disc diffusion 24 h		Allelic variant				
NTHi strain	CT	Patient	MIC	Phenotype	MIC	Phenotype	FtsI	AcrA	AcrB	AcrR	
RdKW20			0.38	S	21	**S**	1	1	1	1	
P667	3	13	1/>32	H2	21	**S**	19	4	12	4	
P668	3	13	1.5/>32	H2	20	**S**	19	4	12	4	
P669	3	13	1/>32	H2	20	**S**	19	4	12	4	
P626	6	7	0.5–0.75	H1	21	**S**	20	14	16	3	
P670	7	13	0.25	S	27	**S**	1	6	17	11	
P672	7	13	0.38	H2	29	**S**	1	6	17	11	
P619	8	6	0.75/>32	H2	20	**H1**	10	8	9	3	
P589	9	1	1.5	H1	21	**H1**	11	9	10	7	
P590	9	1	0.38	H2	25	**H1**	11	9	10	7	
P594	14	2	0.5/>32	H2	25	**H1**	12	10	11	8	
P595	14	2	1	H1	21	**S**	12	10	11	8	
P596	14	22	0.5/2	S	24	**S**	12	10	11	8	
P650	14	10	0.5/4	H1	25	**S**	12	10	11	8	
P676	16	3	0.5	S	24	**S**	19	4	18	4	
P679	16	3	0.5/>32	H2	29	**S**	19	4	18	4	
P853	16	3	1/>32	H2	24	**S**	19	4	18	4	
P627	17	8	0.75	H1	23	**H1**	13	11	12	4	
P628	17	8	0.75/>32	H2	20	**H1**	13	11	12	4	
P641	18	10	2/>32	H2	20	**H2**	6	4	6	4	
P642	18	10	3/>32	H2	20	**H2**	6	4	6	4	
P588	27	1	0.75	H1	22	**H1**	14	4	6	4	
P671	28	13	0.75–1	H2	24	**S**	19	4	12	4	
P610	35	6	0.19	H1	30	**S**	19	4	19	4	
P645	38	10	0.125	S	25	**S**	21	15	20	Truncated	
P607	40	5	0.5/>32	H2	25	**H1**	7	5	7	5	
P608	40	5	0.5/>32	H2	23	**H2**	7	5	7	5	
P617	44	6	6	R	17	**R**	2	2	2	2	
P634	44	9	8	R	17	**R**	3	2	3	2	
P635	44	9	12	R	16	**R**	3	2	3	2	
P636	44	9	3	R	19	**R**	4	2	4	2	
P637	44	9	6	R	19	**R**	4	2	4	2	
P674	45	3	0.5/>32	H2	26	**S**	22	16	21	12	
P675	45	3	0.75/>32	H2	25	**S**	22	16	21	12	
P677	45	3	0.5	S	26	**S**	22	16	21	12	
P678	45	3	0.5/>32	H2	25	**S**	22	16	21	12	
P661	47	12	0.25	S	25	**S**	13	11	12	4	
											**Haplotype**
P600	48	4	0.75/>32	H2	22	**H1**	8	6	Truncated	Truncated	C
P601	48	4	0.75	H1	24	**H1**	8	6	Truncated	Truncated	C
P602	48	4	0.75	H1	21	**S**	8	6	Truncated	Truncated	C
P612	48	6	0.5/>32	H2	24	**H2**	8	6	8	Truncated	C
P613	48	6	0.125–0.19	S	31	**S**	8	6	8	Truncated	B
P614	48	6	0.094	S	30	**S**	8	6	8	Truncated	B
P615	48	6	1.5/>32	H2	22	**H2**	8	6	Truncated	Truncated	C
P616	48	6	1/>32	H2	23	**H2**	8	6	Truncated	Truncated	C
P618	48	6	0.25	S	22	**S**	8	6	8	Truncated	A
P620	48	7	1/>32	H2	21	**H2**	8	6	8	Truncated	D
P621	48	7	0.75/>32	H2	24	**H2**	8	6	8	Truncated	D
P622	48	7	0.5	S	20	**S**	8	6	8	Truncated	D
P623	48	7	0.38/>32	H2	24	**H2**	8	6	8	Truncated	D
P624	48	7	1/>32	H2	20	**H2**	8	6	8	Truncated	D
P629	48	8	0.75/>32	H2	22	**H2**	8	6	8	Truncated	D
P632	48	8	1/>32	H2	25	**S**	8	6	8	Truncated	D
P633	48	8	0.125–0.19	S	31	**S**	8	6	8	Truncated	B
P609	52	5	0.5/>32	H2	22	**S**	15	17	22	6	
P646	54	10	0.5/>32	H2	25	**S**	17	13	23	10	
P647	54	10	0.5/>32	H2	25	**S**	17	13	23	10	
P648	54	10	0.75/>32	H2	25	**S**	17	13	23	10	
P649	54	10	0.25	H1	29	**S**	17	13	23	10	
P597	59	2	0.5	S	22	**S**	23	18	24	13	
P604	72	5	1/>32	H2	22	**H1**	14	4	6	4	
P605	72	5	1/>32	H2	21	**S**	14	4	6	4	
P599	73	4	0.38/>32	H2	21	**H2**	9	7	5	6	
P651	73	10	0.25	H1	23	**H1**	9	7	5	6	
P652	73	10	0.25	H1	21	**H1**	9	7	5	6	
P653	73	10	0.5/>32	H2	20	**H2**	9	7	5	6	
P654	73	10	0.38/>32	H2	20	**H1**	9	7	5	6	
P664	76	12	0.25	S	25,5	**S**	23	18	25	14	
P665	76	12	0.25	S	26	**S**	23	18	25	14	
P666	76	12	0.19	S	22	**S**	23	18	25	14	
P640	83	10	05/>32	H2	20	**H1**	15	7	5	6	
P638	87	10	0.75/>32	H2	20	**H2**	10	8	9	3	
P639	91	10	0.5/>32	H2	27	**H1**	16	12	13	8	
P625	95	7	3/>32	R	17	**R**	5	3	5	3	
P673	95	13	2/>32	H2	21	**H2**	5	3	5	3	
P657	100	11	1	S	24	**S**	23	19	13	Truncated	
P658	100	11	0.75	S	24	**S**	23	19	13	Truncated	
P660	100	11	1.5/>32	H2	20	**S**	23	19	13	Truncated	
P656	105	11	1.5/>32	H2	20	**H1**	17	13	14	9	
P662	106	12	0.38/>32	H2	20	**S**	19	4	12	4	
P663	106	12	0.032–0.047/>32	H2	24	**S**	19	4	12	4	
P591	107	1	3/>32	H2	20	**S**	24	7	26	Truncated	
P603	119	5	0.5/>32	H2	20	**S**	25	20	27	15	
P630	124	8	1.5/>32	H2	20	**H1**	18	4	12	4	
P593	135	2	0.38/>32	H2	25	**H1**	17	13	15	10	
P598	137	4	0.5/>32	H2	26	**S**	17	4	28	4	
P631	137	8	0.38/>32	H2	23	**S**	17	4	28	4	
P611	138	6	1/>32	H2	20	**S**	14	4	25	4	
P851	140	3	0.75	S	22	**S**	26	21	29	Truncated	
P606	145	5	0.5	H1	24	**S**	21	11	12	4	
P592	146	1	0.5	H1	25	**S**	27	21	30	Truncated	

CT: clonal type. Bold text: strain categories established for further study.

**Figure 3:**
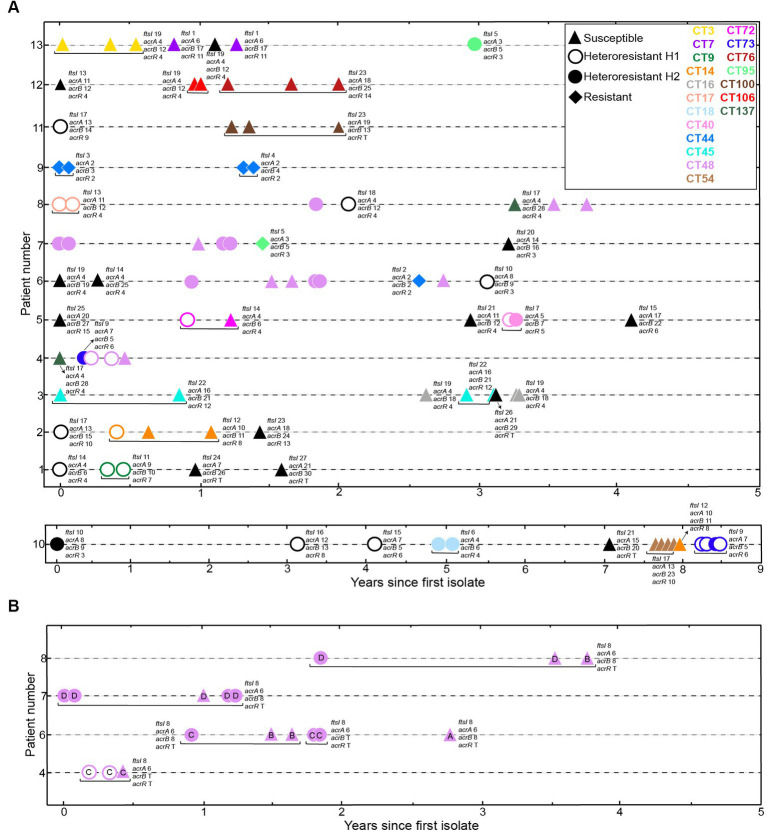
Distribution of imipenem phenotypes in longitudinally sampled NTHi COPD isolates. **(A)** We used 90 NTHi isolates collected over 1 to 9 years from 13 COPD patients (Moleres et al., 2018). Axes indicate patient identifier (ID) numbers and the time of sampling for each isolate, respectively. Symbols refer to each isolate phenotype, based on disc diffusion assay: triangles, susceptible, S; open circles, heteroresistant H1; closed circles, heteroresistant H2; diamonds, resistant, R. Symbol color indicate clonal type (CT), as previously established (Moleres et al., 2018). Black refers to CTs collected only once; yellow, CT3; dark purple (CT7); green (CT9); orange (CT14); grey (CT16); coral pink (CT17); light blue (CT18); pink (CT40); blue (CT44); cyan (CT45); light purple (CT48); light brown (CT54); fuchsia (CT72); dark blue (CT73); deep red (CT76); light green (CT95); dark brown (CT100); red (CT106); dark green (CT137). **(B)** Distribution of strains belonging to CT48, isolated from four patients over time. Strains in CT48, previously classified in four haplotypes, A-D (Moleres et al., 2018), showed intra-haplotype phenotypic variation in haplotypes C and D. Letter inside each symbol refers to haplotype. Distribution of *ftsI*, *acrA*, *acrB* and *acrR* variants is indicated for each strain. Please note that, for strains belonging to CT48, gene variant distribution is indicated in panel (B).

Extensive use of the disc diffusion assay in clinical procedures, in comparison to Etest, led us to use disc diffusion as the method of choice to establish strain categories for further analysis (Table 1, column “Disc diffusion 24 h”, subcolumn “Phenotype”, bold text). Phenotypic variation within patient and/or clonal type (CT) was next analyzed, data are summarized in Figure 3 and Table 1. Within-patient phenotypic variation (susceptible, S; heteroresistant, H; resistant, R) was observed for all patients except for patients 3 and 12 (all isolates were susceptible), and for patient 9 (clonal resistant isolates) (Figure 3). However, when looking at strains belonging to the same CT, phenotypic variation was less common. For clarity, CTs were previously established by applying goeBurst in PHYLOViZ to allele assignments made from core protein-coding genes shared by all genomes. For each single-copy core gene, unique nucleotide sequences were defined as distinct alleles, strains with <15 allelic differences across all core genes were clustered together, and each resulting connected component was assigned an arbitrary CT number (Moleres et al., 2018). In CTs 3, 7, 16, 45, 54, 76, 100, 106 and 137, all isolates were susceptible; in CTs 9, 17, 18, 40 and 73, all isolates were heteroresistant; in CT44, all isolates were resistant. Only CTs 14, 48, 72 and 95 showed intra-CT phenotypic variation. Strains in CTs 40 and 73 showed different degrees of heteroresistance (H1 or H2 phenotypes); strains in CTs 14, 48 and 72 were susceptible or heteroresistant; strains in CT 95 were resistant o heteroresistant (Table 1).

Further heteroresistance characterization assays were carried out (Steps 2 and 3 in Figure 1). Step 2 consisted of collecting the colonies grown within the disc diffusion inhibition zone of heteroresistant strains, to be used as starting inoculum in a new disc diffusion assay. In all cases, the heteroresistance phenotype was maintained. Step 3 consisted of profiling imipenem heteroresistance by the quantitative method of population analysis (Figure 2B). Although considered to be reliable to identify heteroresistance, population analysis profiling (PAP) interpretation may be complex as several aspects are considered when defining this phenotype, including the presence of a resistant subpopulation with a MIC at least eightfold higher than the highest concentration of drug that does not affect the growth of the main population, the growth of the resistant subpopulation above the clinical breakpoint with frequency above 1×10^−7^, or a gradual decrease in the number of CFU instead of a single-step loss of survival (Andersson et al., 2019; Stojowska-swędrzyńska et al., 2022). Here, 19 isolates displaying a heterogeneous resistant profile by the disc diffusion method were selected for this analysis as representative of genomic heterogeneity (7 strains belonging to single-strain CTs, P593, P630, P639, P638, P640, P588 and P619; 12 strains belonging to multi-strain CTs, P653, P652, P604, P623, P616, P612, P600, P608, P607, P628, P594 and P590, see Table 1). RdKW20 was used as a susceptible control. Based on growth at the antibiotic concentrations tested, three strain groups were observed: subpopulations of cells growing on medium containing ≥8XMIC, including concentrations higher than 2 μg/mL imipenem (EUCAST clinical breakpoint), were present in the cultures of strains P612, P623, P653, P652, P640 and P593 (31.57%, 6/19), Figure 2B, left panel; subpopulations of cells growing on medium containing ≥4XMIC, including concentrations higher than 2 μg/mL imipenem were present in the cultures of strains P619, P590, P594, P588, P607, P608, P638, P639 and P630 (47.36%, 9/19), Figure 2B, middle panel; subpopulations of cells growing on medium containing ≥2XMIC with a gradual decrease in the number of CFU were present in the cultures of strains P628, P600, P616 and P604 (21.05%, 4/19), Figure 2B, right panel. H1 and H2 strains were distributed across these three groups. Intra-CT variation was not observed in this assay, strains P652 and P653 (CT73) belong to the >8XMIC group; strains P607 and P608 (CT40) belong to the >4XMIC group. Correlation between disc diffusion and PAP was observed as all tested strains contained subpopulations of cells growing on medium with imipenem concentrations higher than their respective MIC, but strain behavior was heterogeneous and not always fulfilling all PAP heteroresistance recommended criteria.

Lastly, given that the disc diffusion method does not allow detecting antibiotic tolerance, additional testing was considered (Figure 1 step 4). The tolerance disc (TD) test (Gefen et al., 2017) was adapted to *H. influenzae* growth requirements (see Methods section). This method relies on tolerant bacteria surviving the transient exposure to the antibiotic, without growing in the inhibition zone because nutrients are depleted by bacteria growing beyond the inhibition zone; growth of tolerant bacteria can be recovered by replacing the antibiotic disc with a new disc with nutrients, leading to colony growth in the inhibition zone. Imipenem tolerance was TD-tested for the 50 susceptible non-heteroresistant strains present in the COPD collection under study, rendering negative results in all cases.

In sum, under the conditions tested, *H. influenzae* heterogeneous heteroresistance, but not tolerance, was found in a collection of NTHi respiratory isolates. Next, seeking for possible gene to phenotype associations, we analyzed variation both in specific genes and at the genome wide level.

### 2.2 Mutation patterns in the PBP3 and AcrAB-TolC efflux pump components

Previous studies showed that polymorphisms in the *ftsI* gene, encoding the transpeptidase domain of penicillin-binding protein 3 (PBP3), and in the *acrA*, *acrB* and *acrR* genes, encoding AcrAB-TolC efflux pump component and regulatory elements, may associate to imipenem heteroresistance (Cerquetti et al., 2007; Cherkaoui et al., 2017, 2018a,b). We examined those genes allelic variation in the strain collection under study, using RdKW20 sequence as a reference. For the four genes, we refer as variant 1 to the one present in strain RdKW20 (Tables 2–5).

**Table 2:** FtsI allelic variation in the *H. influenzae* strain collection under study.

Allelic variant	Amino acid substitution for:																																						
Rd KW20	S 7	T 21	A 22	P 31	V 34	C 56	A 62	N 75	N 122	L 124	A 125	K 127	A 131	E 135	E 141	S 152	L 165	S 166	R 173	L 219	T 228	A 239	S 273	V 307	G 341	D 350	M 377	A 423	A 437	I 449	G 490	A 502	V 511	R 517	N 526	A 530	V 547	D 551	Phenotype
1	.	.	.	.	.	.	.	.	.	.	.	.	.	.	.	.	.	.	.	.	.	.	.	.	.	.	.	S	.	.	.	.	.	.	.	.	.	.	S
2	.	.	.	S	.	.	.	.	.	.	.	.	.	.	.	.	.	.	.	.	.	.	.	.	.	.	.	R	.	.	.	.	.	.	K	S	I	.	R
3	.	.	.	S	.	G	.	S	H	.	V	.	S	Q	.	A	S	N	.	M	I	E	.	.	.	N	.	R	.	.	.	T	.	.	K	.	I	.	R
4	.	.	.	S	.	.	.	.	.	.	.	.	.	.	K	.	.	.	K	.	.	E	.	.	.	.	.	R	.	.	.	V	.	.	K	.	I	.	R
5	.	.	.	S	.	.	.	.	.	.	.	.	.	.	K	.	.	.	K	.	.	E	.	.	.	.	.	R, H	.	V	.	.	.	.	K	.	I	.	R, H
6	.	.	.	S	.	.	.	.	.	I	.	.	S	Q	.	A	S	N	.	M	I	E	.	.	.	N	.	H	.	.	.	T	.	.	K	.	I	.	H
7	.	.	.	.	.	.	.	.	.	.	.	T	.	.	K	.	.	.	.	.	.	E	.	.	.	.	.	H	.	.	.	.	.	.	.	.	.	.	H
8	.	.	.	S	.	.	.	.	.	.	.	.	.	.	K	.	.	.	K	.	.	E	.	.	.	N	.	H, S	.	.	.	T	.	.	K	.	I	.	H, S
9	.	.	.	S	.	.	.	.	.	.	.	.	.	.	.	.	.	.	.	.	.	.	.	.	.	N	.	H, S	.	.	E	.	.	.	K	S	.	.	H, S
10	.	.	.	S	.	.	.	.	.	.	.	.	.	.	.	.	S	.	.	.	.	E	.	.	.	.	.	H	.	.	.	.	.	.	.	.	.	.	H
11	.	.	.	.	.	.	.	.	.	.	.	.	.	.	.	.	.	.	.	M	I	E	.	.	.	N	.	H	.	.	.	T	.	.	K	.	.	.	H
12	.	.	.	.	.	.	.	.	.	.	.	.	.	.	K	.	.	.	.	.	.	E	.	.	S	.	.	H, S	.	.	.	.	.	.	.	.	.	.	H, S
13	.	.	.	S	.	.	.	.	.	.	.	.	.	.	.	.	.	.	.	.	.	.	.	.	.	.	.	H, S	.	.	.	.	.	.	.	.	I	.	H, S
14	.	.	T	.	.	.	.	.	.	.	.	.	.	.	.	.	.	.	.	.	.	E	.	.	.	.	.	H, S	.	.	.	.	.	.	.	.	.	.	H, S
15	.	.	.	.	.	.	S	.	.	.	.	T	.	.	K	.	.	.	.	.	.	E	.	.	.	.	.	H	.	.	.	.	.	.	.	.	.	.	H
16	.	.	.	.	.	.	.	.	.	.	G	.	.	.	K	.	.	.	.	.	.	E	.	.	.	.	.	H	.	.	.	S	.	.	.	.	.	.	H
17	.	.	.	.	.	.	.	.	.	.	.	.	.	.	.	.	.	.	.	.	.	E	.	.	.	.	.	H, S	.	.	.	.	.	.	.	.	.	.	H, S
18	.	.	.	S	.	G	.	S	H	.	V	.	S	Q	.	A	S	N	.	I	I	E	.	.	.	N	I	H	.	.	E	V	.	.	K	.	I	.	H
19	.	.	.	S	.	.	.	.	.	.	.	.	.	.	K	.	.	.	K	.	.	E	.	.	.	.	.	S	.	.	.	.	.	.	.	.	.	.	S
20	.	.	.	S	.	.	.	.	.	.	G	.	.	.	K	.	.	.	.	.	.	E	.	.	.	.	.	S	.	.	.	.	.	.	.	.	.	.	S
21	.	.	.	S	.	.	.	.	.	.	.	.	.	.	.	.	.	.	.	.	.	.	.	.	.	.	.	S	.	.	.	.	.	.	.	.	.	.	S
22	.	.	.	.	.	.	.	.	.	.	.	.	.	.	.	.	.	.	.	.	.	.	.	.	.	.	.	S	.	.	.	.	.	.	.	.	.	A	S
23	.	.	.	.	.	.	.	.	.	.	.	.	.	.	K	.	.	.	.	.	.	E	.	.	.	.	.	S	.	.	.	.	.	.	.	.	.	.	S
24	.	.	T	.	.	.	.	.	.	.	.	.	.	.	K	.	.	.	.	.	.	E	.	.	.	.	.	S	.	.	.	.	.	.	.	.	.	.	S
25	P	.	.	.	L	.	.	S	.	.	.	.	.	Q	.	A	S	N	.	I	.	E	.	I	.	N	.	S	.	.	.	.	.	.	.	.	I	.	S
26	P	A	.	.	L	.	.	S	.	.	.	.	.	Q	.	A	S	N	.	I	.	E	A	.	.	.	.	S	S	.	.	.	A	H	.	.	I	.	S
27	.	.	.	S	.	.	.	.	.	.	.	.	.	.	.	.	S	.	.	.	.	E	.	.	.	.	.	S	.	.	.	T	.	.	K	.	I	.	S

Allelic variant	Amino acid substitution for:											
Rd KW20	N 569	A 586	A 589	T 591	S 594	A 595	I 601	E 603	V 609	Phenotype	Nº strains	%
1	.	.	.	.	.	.	.	.	.	S	2	2.22
2	.	.	.	.	.	.	L	G	.	R	1	1.11
3	S	.	.	.	.	.	.	D	.	R	2	2.22
4	S	S	.	.	T	T	.	.	.	R	2	2.22
5	S	S	.	.	T	T	.	D	.	R,H	2	2.22
6	S	.	.	.	T	T	.	D	.	H	2	2.22
7	.	.	.	.	.	.	.	.	.	H	2	2.22
8	S	.	.	.	T	T	.	D	.	H,S	17	18.89
9	.	.	.	.	.	.	.	.	.	H,S	6	6.67
10	.	.	.	.	.	.	.	.	.	H	2	2.22
11	.	.	.	.	.	.	.	.	.	H	2	2.22
12	.	.	.	.	.	.	.	D	.	H, S	4	4.44
13	.	.	.	.	.	.	.	.	.	H, S	3	3.33
14	.	.	.	.	.	.	.	D	.	H, S	4	4.44
15	.	S	.	.	.	.	.	.	.	H	1	1.11
16	.	.	.	.	.	.	.	.	.	H	1	1.11
17	.	.	.	.	.	.	.	.	.	H, S	8	8.89
18	S	P	K	A	.	.	V	D	.	H	1	1.11
19	.	.	.	.	.	.	.	.	.	S	10	11.11
20	.	.	.	.	.	.	.	D	.	S	1	1.11
21	.	.	.	.	.	.	.	.	.	S	2	2.22
22	.	.	.	.	.	.	.	.	.	S	4	4.44
23	.	.	.	.	.	.	.	.	.	S	7	7.78
24	.	S	.	.	.	.	.	.	M	S	1	1.11
25	S	.	.	.	.	.	.	N	.	S	1	1.11
26	S	S	.	.	T	T	.	N	.	S	1	1.11
27	.	.	.	.	.	.	.	.	.	S	1	1.11

S7P, T21A, A22T, V34L, A62S, N122H, A125V, A125G, K127T, R173K, V307I, G341S, A423V and V609M changes are newly described in this study.“.” indicates no amino acid substitution. We refer as variant 1 to the one present in reference strain RdKW20.

**Table 3:** AcrA allelic variation in the *H. influenzae* strain collection under study.

Allelic variant	Amino acid substitution for:																																								
Rd KW20	L 16	I 19	V 22	V 29	G 30	M 36	I 37	G 39	V 40	I 42	A 45	G 48	S 52	V 76	P 78	N 79	A 82	M 83	T 86	A 89	A 91	V 92	N 102	E 107	V 108	V 110	S 114	S 115	R 118	A 119	N 120	S 129	V 139	G 140	N 143	V 147	V 163	S 165	A 172	I 191	Phenotype
1	.	.	.	.	.	.	.	.	.	.	.	.	.	.	.	.	.	.	.	.	.	.	.	.	.	.	.	.	.	.	.	.	.	.	.	.	.	S	.	.	S
2	.	.	.	.	.	I	.	.	.	.	.	E	.	.	.	.	.	L	.	.	T	I	.	.	.	.	.	.	.	.	.	.	.	.	.	.	L	R	V	.	R
3	.	.	.	.	.	.	.	.	.	.	.	E	P	.	.	.	V	.	.	.	.	.	.	.	.	.	.	.	.	.	.	.	.	.	.	.	.	R, H	.	V	R, H
4	.	.	.	.	.	I	.	.	.	.	.	E	.	.	.	.	.	L	.	.	T	I	.	.	.	.	.	.	.	.	.	.	.	.	.	.	L	H, S	V	.	H, S
5	.	.	.	.	.	.	.	.	.	.	.	E	.	.	.	.	.	.	.	.	T	I	Q	D	L	I	.	.	H	.	S	P	A	N	K	.	L	H	V	V	H
6	.	.	.	.	.	.	.	.	.	.	.	.	P	I	.	H	.	.	A	.	T	.	.	.	.	.	.	.	.	.	S	.	.	S	.	.	.	H, S	V	.	H, S
7	.	.	.	A	S	.	.	.	I	.	.	E	P	.	.	.	.	.	.	T	.	.	.	.	.	.	.	.	.	.	.	.	.	.	.	.	.	H, S	.	V	H, S
8	.	.	.	.	.	.	.	.	.	.	.	E	P	.	.	.	V	.	.	.	.	.	.	.	.	.	.	.	.	.	.	.	.	.	.	.	.	H	.	V	H
9	.	V	A	A	.	V	F	.	.	T	V	S	P	I	.	H	.	.	A	.	T	.	.	.	.	.	N	F	.	.	S	.	.	S	.	.	.	H	.	.	H
10	.	.	.	.	.	.	.	.	I	.	.	E	P	.	.	.	.	.	.	.	.	.	.	.	.	.	.	.	.	.	.	.	.	.	.	.	.	H, S	.	V	H, S
11	.	.	.	.	.	I	.	.	.	.	.	E	.	.	.	.	.	L	.	.	T	I	.	.	.	.	.	.	.	.	.	.	.	.	.	.	L	H, S	V	.	H, S
12	.	.	.	.	.	.	.	.	.	.	.	E	P	.	.	.	.	.	.	.	.	.	.	.	.	.	.	.	.	.	.	.	.	.	.	.	.	H	.	V	H
13	F	.	.	.	.	.	.	.	.	.	.	.	P	.	A	.	.	.	.	.	T	I	.	D	L	I	.	.	H	.	S	P	A	N	K	.	L	H, S	V	V	H, S
14	.	.	.	.	.	.	.	.	.	.	.	E	P	.	A	.	.	.	.	.	T	I	Q	D	L	I	.	.	H	.	S	P	A	N	K	.	L	S	V	V	S
15	.	.	.	.	.	.	.	.	.	.	.	E	P	.	.	.	.	.	.	.	T	I	.	.	.	.	.	.	.	S	.	.	.	.	.	.	.	S	.	.	S
16	F	.	.	.	.	.	.	.	.	.	.	E	P	.	.	.	.	.	.	.	.	.	.	.	.	.	.	.	.	.	.	.	.	.	.	.	.	S	.	V	S
17	.	.	.	.	.	.	.	.	.	.	.	E	P	.	.	.	.	.	.	.	.	.	Q	D	L	I	.	.	H	.	S	P	A	N	K	I	L	S	V	V	S
18	F	.	.	.	.	.	.	.	.	.	.	E	P	.	.	.	.	.	.	.	.	.	.	.	.	.	.	.	.	.	.	.	.	.	.	.	.	S	.	V	S
19	.	.	.	A	S	.	.	.	I	.	.	E	P	.	.	.	.	.	.	.	.	.	.	.	.	.	.	.	.	.	.	.	.	.	.	.	.	S	.	.	S
20	.	V	A	A	.	I	F	.	.	T	V	S	P	I	.	H	.	.	A	.	T	.	.	.	.	.	.	.	.	.	S	.	.	S	.	.	.	S	V	.	S
21	F	.	.	.	.	.	.	D	.	.	.	E	P	.	A	.	.	.	.	.	T	I	Q	D	L	I	.	.	H	.	S	P	A	N	K	.	.	S	.	V	S

Allelic variant	Amino acid substitution for:																												
Rd KW20	V 193	G 229	T 236	L 240	E 242	I 255	S 257	S 258	V 264	D 269	P 270	G 273	H 274	P 289	V 295	V 296	E 315	E 319	E 321	S 326	D 332	R 333	Y 351	V 361	D 385	I 390	Phenotype	N° strains	%
1	.	.	.	.	.	.	.	.	.	.	.	.	.	.	.	.	.	.	.	.	.	.	.	.	.	.	S	0	0
2	.	.	.	.	K	.	.	.	.	.	.	.	.	.	.	.	.	.	.	.	.	.	.	A	.	V	R	5	5.56
3	.	.	.	.	.	.	.	.	.	.	.	.	.	.	.	.	.	.	.	.	.	.	.	A	.	.	R, H	2	2.22
4	.	.	.	.	.	.	.	.	.	N	.	.	.	.	.	.	.	.	.	.	.	.	.	A	.	V	H, S	19	21.11
5	.	.	.	.	.	.	.	.	.	.	.	.	.	.	.	.	.	.	.	L	.	.	.	A	.	V	H	2	2.22
6	I	S	V	S	.	V	A	A	I	.	.	E	R	S	I	.	T	D	D	.	.	.	.	A	.	V	H, S	19	21.22
7	.	.	.	.	.	.	.	.	.	.	.	.	.	.	.	I	.	.	.	.	.	H	.	A	.	.	H, S	7	7.78
8	.	.	.	.	.	.	.	.	.	.	.	.	.	.	.	.	.	.	.	.	.	.	.	A	.	V	H	2	2.22
9	I	.	V	S	.	V	A	A	I	.	.	.	R	S	I	.	T	D	D	.	.	.	.	A	.	V	H	2	2.22
10	.	.	.	.	.	.	.	.	.	.	S	.	.	.	.	.	.	.	.	.	.	.	H	A	.	V	H, S	4	4.44
11	.	.	.	.	.	.	.	.	.	N	.	.	.	.	.	.	.	.	.	.	.	.	.	A	G	V	H, S	4	4.44
12	.	.	.	.	.	.	.	.	.	.	.	.	.	.	.	.	.	.	.	.	.	.	.	A	.	V	H	1	1.11
13	.	.	.	.	.	.	.	.	.	.	.	.	.	.	.	.	.	.	.	.	.	.	.	A	.	V	H, S	6	6.67
14	.	.	.	.	.	.	.	.	.	N	.	.	.	.	.	.	.	.	.	.	N	.	.	A	.	V	S	1	1.11
15	.	.	.	.	.	.	.	.	.	.	.	.	.	.	.	.	.	.	.	.	.	.	.	A	.	V	S	1	1.11
16	.	.	.	.	K	.	.	.	.	.	.	.	.	.	.	.	.	.	.	.	.	.	.	A	.	V	S	4	4.44
17	.	.	.	.	.	.	.	.	.	.	.	.	.	.	.	.	.	.	.	.	.	.	.	A	.	V	S	1	1.11
18	.	.	.	.	.	.	.	.	.	.	.	.	.	L	.	.	.	.	.	.	.	.	.	A	.	V	S	4	4.44
19	.	.	.	.	.	.	.	.	.	.	.	.	.	.	.	.	.	.	.	.	.	.	.	A	.	V	S	3	3.33
20	I	.	V	S	.	V	A	A	I	.	.	.	R	.	.	.	T	D	D	.	.	.	.	A	.	V	S	1	1.11
21	.	.	.	.	.	.	.	.	.	.	.	.	.	.	.	.	.	.	.	.	.	.	.	A	.	V	S	2	2.22

L16F, I19V, V22A, V29A, G30S, M36V, I37F, G39D, V40I, I42T, A45V, V76I, P78A, N79H, T86A, A89T, N102Q, E107D, V108L, V110I, S114N, S115F, R118H, A119S, N120S, S129P, V139A, G140N, G140S, N143K, V147I, A172V, V193I, G229S, T236V, L240S, E242K, I255V, S257A, S258A, V264I, P270S, G273E, H274R, P289S, P289L, V295I, E315T, E319D, E321D, S326L, D332N, Y351H and D385G are newly described in this study. “.” indicates no amino acid substitution. We refer as variant 1 to the one present in reference strain RdKW20.

**Table 4:** AcrB allelic variation in the *H. influenzae* strain collection under study.

Allelic variant	Amino acid substitution for:																																									
Rd KW20	V 12	F 164	V 199	T 201	K 234	N 266	M 373	I 408	A 414	T 426	V 474	A 492	K 493	M 497	E 499	R 500	V 511	- 512	- 513	- 514	- 515	- 516	- 517	- 518	- 519	- 520	- 521	- 522	V 542	S 554	E 596	T 611	S 614	S 628	A 633	I 634	I 638	E 640	A 642	I 655	I 658	Phenotype
1	.	.	.	.	.	.	.	.	.	.	.	.	.	.	.	.	.	-	-	-	-	-	-	-	-	-	-	-	.	.	.	.	.	.	.	.	.	.	.	.	.	S
2	.	.	.	.	.	S	.	V	E	.	I	.	Q	L	Q	K	I	-	-	-	-	-	-	-	-	-	-	-	.	A	D	N	G	T	V	V	L	A	.	F	.	R
3	.	.	.	.	.	S	.	.	.	.	.	.	.	.	.	.	.	-	-	-	-	-	-	-	-	-	-	-	.	.	.	.	.	.	.	.	.	.	.	.	.	R
4	.	.	.	.	.	S	.	.	.	.	.	.	.	.	.	.	.	-	-	-	-	-	-	-	-	-	-	-	.	.	.	.	.	.	.	.	.	.	.	.	.	R
5	.	.	.	.	.	.	.	.	.	V	.	.	.	.	.	.	.	-	-	-	-	-	-	-	-	-	-	-	.	.	.	.	.	.	.	.	.	.	.	.	.	R, H
6	.	.	.	.	.	.	.	V	.	.	I	.	.	.	.	.	.	-	-	-	-	-	-	-	-	-	-	-	.	A	.	N	G	.	.	.	.	.	.	.	.	H, S
7	.	.	.	.	.	.	.	.	.	.	.	.	.	.	.	.	.	-	-	-	-	-	-	-	-	-	-	-	.	.	.	.	.	.	.	.	.	.	.	.	.	H
8	.	.	.	.	.	.	.	.	.	.	.	.	.	.	.	.	.	-	-	-	-	-	-	-	-	-	-	-	.	.	.	.	.	.	.	.	.	.	.	.	.	H, S
9	.	.	.	.	.	S	.	.	.	.	.	.	.	.	.	.	.	-	-	-	-	-	-	-	-	-	-	-	.	.	.	.	.	.	.	.	.	.	.	.	.	H
10	.	.	A	A	T	S	.	V	E	.	I	.	Q	L	Q	K	I	-	-	-	-	-	-	-	-	-	-	-	.	A	D	N	G	T	V	V	L	A	.	F	.	H
11	.	.	.	.	.	S	I	.	.	.	I	.	.	.	.	.	.	-	-	-	-	-	-	-	-	-	-	-	.	.	.	.	.	.	.	.	.	.	.	.	.	H, S
12	.	.	.	.	.	.	.	.	.	.	.	.	.	.	.	.	.	-	-	-	-	-	-	-	-	-	-	-	.	.	.	.	.	.	.	.	.	.	.	.	.	H, S
13	.	.	.	.	.	S	.	.	.	.	.	.	.	.	.	.	.	-	-	-	-	-	-	-	-	-	-	-	.	.	.	.	.	.	.	.	.	.	.	.	.	H, S
14	.	.	.	.	.	.	.	.	.	.	.	T	.	.	.	.	.	-	-	-	-	-	-	-	-	-	-	-	.	.	.	.	.	.	.	.	.	.	.	.	.	H
15	I	.	.	.	.	.	.	.	.	.	.	.	.	.	.	.	.	-	-	-	-	-	-	-	-	-	-	-	.	.	.	.	.	.	.	.	.	.	.	.	.	H
16	.	.	.	.	.	.	.	.	.	.	.	.	.	.	.	.	.	-	-	-	-	-	-	-	-	-	-	-	.	.	.	.	.	.	.	.	.	.	.	.	.	S
17	.	.	.	.	.	.	.	.	.	.	.	.	.	.	.	.	.	-	-	-	-	-	-	-	-	-	-	-	.	.	.	.	.	.	.	.	.	.	.	.	.	S
18	.	.	.	.	.	.	.	.	.	.	.	.	.	.	.	.	.	-	-	-	-	-	-	-	-	-	-	-	.	.	.	.	.	.	.	.	.	.	.	.	.	S
19	.	.	.	.	.	.	.	.	.	.	.	.	.	.	.	.	.	-	-	-	-	-	-	-	-	-	-	-	.	.	.	.	.	.	.	.	.	.	.	.	.	S
20	.	.	.	.	.	.	.	.	.	.	.	.	.	.	.	.	.	-	-	-	-	-	-	-	-	-	-	-	.	.	.	.	.	.	.	.	.	.	.	.	.	S
21	.	L	.	.	.	.	.	.	.	.	.	.	.	.	.	.	.	-	-	-	-	-	-	-	-	-	-	-	.	.	.	.	.	.	.	.	.	.	.	.	.	S
22	.	.	.	.	.	.	.	.	.	V	.	.	.	.	.	.	.	-	-	-	-	-	-	-	-	-	-	-	.	.	.	.	.	.	.	.	.	.	.	.	.	S
23	.	.	.	.	.	.	.	.	.	.	.	.	.	.	.	.	.	-	-	-	-	-	-	-	-	-	-	-	.	.	.	.	.	.	.	.	.	.	.	.	.	S
24	.	L	.	.	.	S	.	.	.	.	.	.	.	.	.	.	.	-	-	-	-	-	-	-	-	-	-	-	.	.	.	.	.	.	.	.	.	.	.	.	.	S
25	.	.	.	.	.	S	.	.	.	.	.	.	.	.	.	.	.	-	-	-	-	-	-	-	-	-	-	-	.	.	.	.	.	.	.	.	.	.	G	.	.	S
26	.	.	.	.	.	.	.	.	.	A	.	.	.	.	.	.	.	V	E	H	T	L	G	K	V	N	R	V	.	.	.	.	.	.	.	.	.	.	.	.	.	S
27	.	.	A	A	T	S	.	V	E	.	I	.	Q	L	Q	K	I	-	-	-	-	-	-	-	-	-	-	-	A	A	D	N	G	T	V	V	L	A	.	F	V	S
28	.	.	.	.	.	S	.	.	.	.	.	.	.	.	.	.	.	-	-	-	-	-	-	-	-	-	-	-	.	.	.	.	.	.	.	.	.	.	.	.	.	S
29	.	.	.	.	.	S	.	.	.	.	I	.	.	.	.	.	.	-	-	-	-	-	-	-	-	-	-	-	.	.	.	.	.	.	.	.	.	.	.	.	.	S
30	.	.	.	.	.	S	.	.	.	.	I	.	.	.	.	.	.	-	-	-	-	-	-	-	-	-	-	-	.	.	.	.	.	.	.	.	.	.	.	.	.	S

Allelic variant	Amino acid substitution for:																															
Rd KW20	P 665	S 689	T 700	D 703	T 705	R 745	Q 781	T 798	I 819	Q 831	T 833	F 842	A 859	V 860	A 863	I 867	M 887	I 903	S 908	I 909	Q 919	L 945	H 947	L 981	I 949	I 996	V 1,020	K 1,034	T 1,035	Phenotype	Nº strains	%
1	.	.	.	.	.	.	.	.	.	.	.	.	.	.	.	.	.	.	.	.	.	.	.	.	.	.	.	.	.	S	0	0
2	.	N	S	N	.	.	.	.	T	.	N	Y	T	T	I	V	.	.	.	.	.	.	.	.	.	.	.	.	.	R	1	1.11
3	A	.	.	.	.	.	.	.	T	H	.	.	.	.	.	.	.	.	.	.	.	.	.	.	M	.	.	.	A	R	2	2.22
4	A	.	.	.	.	.	.	.	T	H	.	.	.	.	.	.	.	.	.	.	.	.	.	.	.	.	.	.	.	R	2	2.22
5	.	.	.	.	.	.	.	.	T	.	N	Y	T	T	I	V	.	.	.	.	.	.	Y	.	.	.	.	.	.	R, H	8	8.89
6	A	.	.	.	.	.	.	.	T	.	N	Y	T	T	I	V	.	L	G	.	K	.	Y	.	M	.	.	.	.	H, S	6	6.67
7	A	.	.	.	.	.	.	.	T	.	N	Y	T	T	I	V	.	.	.	.	.	.	Y	.	.	.	.	.	.	H	2	2.22
8	A	.	.	.	.	.	.	.	.	.	N	Y	T	T	I	V	.		.	.	.	.	.	.	.	.	.	.	.	H, S	12	13.33
9	.	.	.	.	.	.	.	.	T	.	N	Y	T	T	I	V	.	L	G	M	K	.	Y	.	M	V	.	.	.	H	2	2.22
10	.	N	S	N	.	.	.	.	T	.	N	Y	T	T	I	V	.	L	G	.	K	.	Y	.	M	.	.	.	.	H	2	2.22
11	A	.	.	.	.	.	.	.	T	H	.	.	.	.	.	.	.	.	.	.	.	.	Y	.	.	.	.	.	.	H, S	4	4.44
12	A	.	.	.	.	.	.	.	.	.	N	Y	T	T	I	V	.	.	.	.	.	.	Y	.	.	.	I	.	.	H, S	11	12.22
13	A	.	.	.	.	.	.	.	T	H	.	.	.	.	.	.	.	.	.	.	.	.	Y	.	.	.	.	.	.	H, S	4	4.44
14	A	.	.	.	.	.	.	.	.	.	N	Y	T	T	I	V	.	.	.	.	.	.	Y	.	.	.	.	.	.	H	1	1.11
15	A	.	.	.	S	.	.	.	T	.	N	Y	T	T	I	V	.	.	.	.	.	I	.	.	.	.	.	.	.	H	1	1.11
16	A	.	.	.	.	.	.	.	T	H	.	.	.	.	.	.	.	.	.	.	.	.	.	.	.	.	.	.	.	S	1	1.11
17	.	.	.	.	.	.	.	.	T	.	N	Y	T	T	I	V	.	.	.	.	.	.	.	.	.	.	.	.	.	S	2	2.22
18	T	.	.	.	.	.	.	.	.	.	N	Y	T	T	I	V	.	.	.	.	.	.	Y	.	.	.	I	.	.	S	3	3.33
19	A	.	.	.	.	.	.	.	.	.	N	Y	T	T	I	V	.	.	.	.	.	.	Y	I	.	.	I	.	.	S	1	1.11
20	.	.	.	.	S	.	R	.	T	.	N	Y	T	T	I	V	.	.	.	.	.	.	Y	.	.	.	.	.	.	S	1	1.11
21	.	.	.	.	.	.	.	.	T	.	N	Y	T	T	I	V	.	.	.	.	.	.	.	.	.	.	.	.	.	S	4	4.44
22	.	.	.	.	.	.	.	.	T	H	.	.	.	.	.	.	.	.	.	.	.	.	-	.	.	.	I	.	.	S	1	1.11
23	A	.	.	.	S	.	.	.	T	.	N	Y	T	T	I	V	.	.	.	.	.	.	Y	.	.	.	.	.	.	S	4	4.44
24	A	.	.	.	.	.	.	.	T	H	.	.	.	.	.	.	.	.	.	.	.	.	Y	.	.	.	.	.	.	S	1	1.11
25	A	.	.	.	.	H	.	.	T	H	.	.	.	.	.	.	.	.	.	.	.	.	.	.	.	.	.	.	.	S	3	3.33
26	.	.	.	.	.	.	.	.	T	.	N	Y	T	T	I	V	.	.	.	.	.	.	Y	.	.	.	.	.	.	S	1	1.11
27	.	N	S	N	.	.	.	.	T	.	N	Y	T	T	I	V	.	L	G	.	K	.	Y	.	M	.	.	.	.	S	1	1.11
28	A	.	.	.	.	.	.	I	T	H	.	.	T	T	I	V	I	.	.	.	.	.	Y	.	.	.	I	E	.	S	2	2.22
29	A	.	.	.	.	.	.	.	T	.	N	Y	T	T	I	V	.	L	G	.	K	.	Y	.	M	.	.	.	.	S	1	1.11
30	A	.	.	.	.	.	.	.	T	H	.	.	.	.	.	.	.	.	.	.	.	.	.	.	.	.	.	.	.	S	1	1.11

“.” indicates no amino acid substitution. We refer as variant 1 to the one present in reference strain RdKW20.

**Table 5:** AcrR allelic variation in the *H. influenzae* strain collection under study.

Allelic variant	Amino acid substitution for:																				
Rd KW20	I 12	S 14	R 18	R 22	N 26	Q 27	L 31	L 33	K 35	T 77	S 110	I 121	H 131	Q 134	L 138	S 143	A 159	K 170	Phenotype	N° strains	%
1	.	.	.	.	.	.	.	.	.	.	.	.	.	.	.	.	.	.	S	0	0
2	.	.	.	.	.	.	H	.	.	.	.	V	D	K	.	.	.	.	R	5	5.56
3	.	.	.	.	.	.	H	.	.	.	.	.	.	.	.	.	.	.	R, H, S	5	5.56
4	.	L	.	K	D	R	H	I	.	S	.	V	D	K	.	.	.	.	H, S	23	25.56
5	.	.	.	K	.	.	H	.	.	.	P	.	.	.	.	.	.	.	H	2	2.22
6	.	.	.	.	.	.	H	.	.	.	.	V	.	.	.	.	.	.	H, S	7	7.78
7	.	L	.	K	D	R	H	I	.	S	.	V	D	K	.	P	V	N	H	2	2.22
8	.	.	.	K	.	.	H	.	.	.	.	.	.	.	.	.	.	.	H, S	5	5.56
9	S	.	.	K	.	.	H	.	.	.	.	V	.	.	.	.	.	.	H	1	1.11
10	.	.	.	K	.	.	H	.	.	.	.	V	.	.	.	.	.	.	H, S	5	5.56
11	.	.	.	K	.	.	H	.	.	.	.	.	.	.	V	.	.	.	S	2	2.22
12	.	.	.	.	.	.	H	.	.	.	.	V	.	K	.	.	.	.	S	4	4.44
13	.	.	.	.	.	.	H	.	N	.	.	V	.	K	.	.	.	.	S	1	1.11
14	.	.	G	.	.	.	H	.	.	.	.	.	.	.	.	.	.	.	S	3	3.33
15	.	L	.	K	D	R	H	I	.	S	.	V	D	K	.	P	.	N	S	1	73.33

“.” indicates no amino acid substitution. We refer as variant 1 to the one present in reference strain RdKW20. I12S, R18G and K35N changes are newly described in this study.

The *ftsI* gene rendered 27 different allelic variants, with 13 newly described amino acid substitutions (Table 2). Regarding variant distribution, 7 variants were found only in heteroresistant strains. Such variants contained, among others, two newly described (Ala62Ser and Lys127Thr) and five previously described (Leu124Ile, Met377Ile, Asn589Lys, The591Ala and Ile601Val) amino acid substitutions that were found only in heteroresistant strains (Table 6).

**Table 6:** Amino acid changes in FtsI, AcrA, AcrB, and AcrR that were only found in imipenem heteroresistant *H. influenzae* isolates.

FtsI			AcrA			AcrB			AcrR		
Amino acid substitutions	N° strains	CT	Amino acid substitutions	N° strains	CT	Amino acid substitutions	N° strains	CT	Amino acid substitutions	N° strains	CT
A62S	1	83	M36V	2	9	V12I	1	135	I12S	1	105
L124I	2	18	S114N	2	9	A492T	1	105	S110P	2	40
K127T	3	40, 83	S115F	2	9	I909M	2	8, 87	A159V	2	9
M377I	1	124	S326L	2	40	L945I	1	135			
A589K	1	124				I996V	2	8, 87			
T591T	1	124									
I601V	1	124									

Allelic variation of the *acrA* and *acrB* genes rendered 20 and 29 different variants, respectively (Tables 3, 4). Please note that AcrA variant 1 and AcrB variant 1 were not present in the strain collection under study. From those, 4 AcrA and 5 AcrB variants were only present in heteroresistant strains. Regarding amino acid changes in AcrA, 51 are newly described, and the Met36Val, Ser114Asn, Ser115Phe, and Ser326Leu changes were only found in heteroresistant strains (Tables 3, 6). Likewise, the amino acid changes Val12Ile, Ala492Thr, Ile909Met, Leu945Ile and Ile996Val in AcrB were only found in heteroresistant strains (Tables 4, 6). AcrB allelic variation also involved loss of function due to a 7-nt insertion (5′-ACTATAT) leading to a truncated variant present in 5 isolates, with susceptible or heteroresistant phenotypes (Supplementary Table S1).

**Figure 4:**
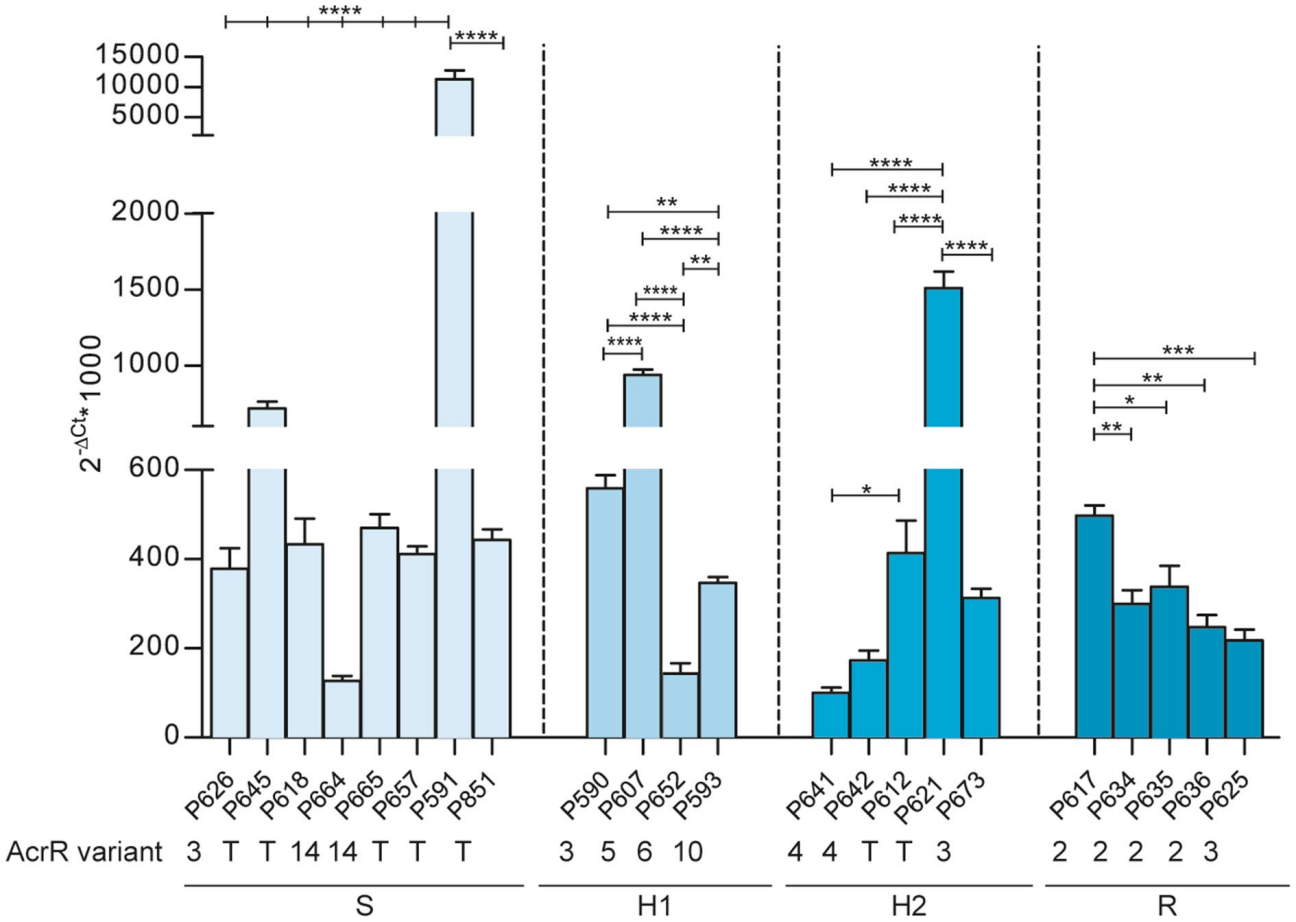
Expression of the *arcA* gene across NTHi respiratory isolates. Expression analysis, determined by RT-qPCR, of the *acrA* gene in 22 strains with truncated (T) or non-truncated *acrR* gene variants (AcrR variant number is indicated). Strains are grouped according to their respective imipenem phenotype (S, susceptible; H1 and H2, heteroresistant; R, resistant). Statistical comparisons of means were performed for each group by one-way ANOVA and Tukey’s multiple-comparison test (*p < 0.05; **p < 0.01; ***p < 0.001; ****p < 0.0001).

The *acrAB* gene expression is negatively regulated by AcrR (Dean et al., 2005), which also undergoes allelic variation. AcrR variation involved amino acid substitutions leading to 14 variants with 3 newly described changes (Table 5). Please note that AcrR variant 1 was not present in the strain collection under study. Three AcrR allelic variants, represented by the Ile12Ser, Ser110Pro and Ala159Val changes, were only present in heteroresistant strains (Table 6). Moreover, AcrR loss of function was found in 10 imipenem heteroresistant and 14 susceptible strains. Truncated variants were due to (i) a C-T transition in three different positions (P645, nucleotide 291; P591, nucleotide 4; P851 and P592, nucleotide 7) rendering an early stop codon in 4 strains; (ii) an AT insertion generating the same frameshift in 17 strains; (iii) three combined 138, 6 and 5 bp deletions in 3 strains (Supplementary Table S2). We next tested expression of the *acrA* gene across strains, considering both *acrR* allelic variation and strain phenotypic heterogeneity for strain set selection. As shown in Figure 4, the *acrA* gene highest expression was observed in strains with AcrR truncated variants (P591, P645, P621), but such gene inactivation did not necessarily lead to high *acrA* gene expression, and full-length variants led in some cases to high *acrA* gene expression (P607). Conversely, phenotypic clustering was not observed, as susceptible (S), heteroresistant (H1 or H2) or resistant (R) phenotypes did not correlate with *acrR* allelic variation and/or with *acrA* gene expression. Therefore, although we do not exclude an association to imipenem heteroresistance, the AcrAB-TolC efflux pump component and regulatory elements are not only contributors to the observed phenotypes.

The *ftsI*, *acrA*, *acrB* and *acrR* gene allelic variation rendered a repertoire of variants and amino acid substitutions summarized in Supplementary Table S3. Although maybe contributors, this may be a multifactorial phenotype requiring combinatorial variation. Variation of the *ftsI*, *acrA*, *acrB* and *acrR* genes rendered 38 combined allelic variants. From those, 10 were found only in heteroresistant strains (Supplementary Table S3, highlighted in green). When looking at gene and amino acid variants within those 10 combinations, 9 variants of the *ftsI* gene were found, 4 of them containing at least one of previously mentioned heteroresistance-associated substitutions: FtsI variant 6, Leu124Ile; variant 7, Lys127Thr; variant 15, Ala62Ser, Lys127Thr; variant 18, Met377Ile, Asn589Lys, The591Ala, Ile601Val. Also, 7 *acrA* gene variants were found, 3 of them containing at least one of previously mentioned heteroresistance-associated substitutions: AcrA variant 4, Met36Val; variant 5, Ser326Leu; variant 9, Met36Val, Ser114Asn, Ser115Phe. Nine variants of the *acrB* gene were found, 3 of them with at least one of previously mentioned heteroresistance-associated substitutions: AcrB variant 9, Ile909Met, Ile996Val; variant 14, Ala492Thr; variant 15, Val12Ile, Leu945Ile. Lastly, we found 8 variants of the *acrR* gene, 3 of them with at least one of previously mentioned heteroresistance substitutions: AcrR variant 5, Ser110Pro; variant 7, Ala159Val; variant 9, Ile12Ser. This combined analysis leads us to present a matrix of FtsI, AcrA, AcrB and AcrR variants and SNPs only present in heteroresistant strains (Supplementary Table S3, highlighted in green).

### 2.3 Genomic variation across strain collection does not render gene to heteroresistant phenotype association

Next, taking advantage of this longitudinal set, we analyzed genomic variation among closely related strains, i.e., belonging to the same CT, showing intra-CT phenotypic heterogeneity in terms of imipenem effects. We selected four strain series, belonging to CTs 14, 48, 72 and 95 to examine intra-CT phenotypic variation involved in susceptible-heteroresistant, or heteroresistant-resistant changes. Among all SNPs detected along whole genome alignment, none of them did segregate susceptible and heteroresistant strains. Lastly, a pangenomic analysis was performed to look for differences in present and absent genes between phenotypically different strains. Such analysis did not reveal any candidate gene that was significantly present or absent in susceptible or heteroresistant strains explaining this phenotype. Together, genomic variation across this strain collection did not render gene to phenotype clear-cut associations.

## 3 Discussion

The carbapenems (e.g., imipenem) are a group of antibiotics that belong to the β-lactam class. Low toxicity and low prevalence of resistance make carbapenems a valuable alternative for initial empirical treatment of severe infections before a definitive microbiological diagnosis is available. Imipenem is not a first-line agent against NTHi infections and comprehensive data on imipenem resistance are scarce, but imipenem heteroresistance has been reported in NTHi isolates from an epidemiologically undefined collection of isolates (Cerquetti et al., 2007; Cherkaoui et al., 2017, 2018a,b; Lâm et al., 2020). Following this notion, this study reports a high prevalence rate of imipenem heteroresistance in a longitudinal set of respiratory *H. influenzae* strains. Strains were isolated from COPD sputum samples recovered over time, i.e., persistent isolates from patients which, based on available clinical data, had not been administered imipenem (Moleres et al., 2018), thus excluding a direct correlation between the observed heteroresistance and imipenem exposure. This may be important as previous work by our group showed acquisition of macrolide resistance in *H. influenzae* during persistent respiratory infection in COPD patients receiving long-term azithromycin treatment (Carrera-Salinas et al., 2023), maybe masking possible heteroresistance development. Also, no specific trends were observed among serially isolated and clonally related strains. When looking at strains belonging to the same CT, only four of them showed intra-CT phenotypic variation, i.e., CTs 14, 48 (see haplotypes C and D), 72 and 95.

In terms of methods, differences between Etest and disc diffusion assays were observed. Such differences were most likely due to the different way the antibiotic diffuses into the agar, but strains with heteroresistance by disc diffusion were also heteroresistant in the Etest assay. This, added to the extensive use of the disc diffusion assay in clinical procedures, led us to use it as the method of choice to establish strain categories in this study, and we strongly suggest that these differences should be considered in the clinical practice. Another aspect worth discussing is the heterogeneity observed in terms of heteroresistance intensity. We established two categories (H1 and H2), but it should be emphasized that this phenotypic visual interpretation requires training and should be made by double-blind assessment. Special attention should also be paid to procedures and interpretation of quantitative methods, i.e., population analysis profiling (PAP), as NTHi heterogeneity among strains may hinder inocula normalization when multiple unrelated strains are simultaneously assayed. This aspect was considered here, and it should apply to any phenotypic assay systematically performed with any NTHi clinical strain series. We acknowledge that, for simplicity when performing the PAP assay, the same range of imipenem concentrations was used for all strains independently of their respective MIC, and subpopulations with an MIC x-fold higher that the highest antibiotic dose not affecting the growth of the main population were calculated afterwards. This is another aspect to consider when testing a wide strain number, as adjusting imipenem range to each respective MIC will increase assay complexity. In this regard, performing population analysis profiling in the clinical practice is unlikely as it is time consuming and requires trained personnel. However, heteroresistance frequency should not be eluded in clinical settings and methodical and thorough disc diffusion assay interpretation may contribute to do so. Following this notion and also aiming for practicality, TD-test implementation in the clinical routine may be worth considering, as hardly extra-material and manipulation are required upon disc diffusion, and it may provide key qualitative assessment of tolerance or persistence.

Here, we also tried to identify genomic traits that may account for the observed heteroresistance, but definitive conclusions were not reached. We used a bottom-up approach, addressing allelic variation in a small set of four genes previously associated to *H. influenzae* imipenem heteroresistance, followed by genome-wide allelic variation in available series of clonally related and phenotypically heterogeneous strains and, lastly, genome-wide differences in terms of gene distribution by sorting strains in two groups based on phenotyping, i.e., imipenem susceptible and heteroresistant isolates. The *ftsI*, *arcA*, *arcB* and *arcR* gene allelic variation is synthesized Table 6 by highlighting changes found only in heteroresistant strains, but overall variation is complex to read as shown in Tables 2–5. In the case of the *ftsI* gene encoding PBP3, Asn526Lys and Arg517His amino acid changes were previously associated to imipenem resistance (Cherkaoui et al., 2017), but also appeared in susceptible strains from our collection. Thirty-eight strains (42.2%, 38/90) had changes surrounding the Lys512-Thr-Gly (KTG) motif, being Ala502Thr (20.67%, 24/90), Asn526Lys (40%, 36/90) and Ala530Ser (7.78%, 7/90) the most commonly found. These changes were found across phenotypes. We also found the Met377Ile substitution, surrounding the Ser379-Ser-Asn (SSN) motif, in a heteroresistant strain, and four changes surrounding the Ser327-Thr-Val-Lys (STVK) motif in 36 strains, the most common Asp350Asn (34.44%, 31/90), unlikely related to heteroresistance as found across phenotypes. Conversely, *acrB* inactivation could lead to higher susceptibility; however, this did not seem to be the case as four strains carrying *acrB* truncated variants showed heteroresistance. Also, inactivation of AcrR could lead to higher *acrAB* gene expression and therefore, to higher drug efflux capacity and lower susceptibility; however, this was not necessarily the case as *arcA* gene expression was not necessarily higher in strains presenting *acrR* truncated variants, and 14 out of the 24 strains carrying *acrR* truncated variants showed imipenem susceptibility.

Heteroresistance is common for several different bacterial species and antibiotic classes, but it is often difficult to detect and study due to its phenotypic and genetic instability, and pose clinical concern as it could increase in frequency during antibiotic exposure and cause treatment failure (Andersson et al., 2019). Besides *H. influenzae* (Cerquetti et al., 2007, 2017), heteroresistance to carbapenems has been detected in clinical isolates of *Acinetobacter baumannii, Enterobacter cloacae, Klebsiella pneumoniae, Escherichia coli*, *Pseudomonas aeruginosa*, and *Salmonella typhimurium* (Andersson et al., 2019; Stojowska-swędrzyńska et al., 2022). In terms of mechanisms, resistance mutations (SNPs, insertions and deletions) and increased copy number of tandem amplifications of genes that increase antibiotic resistance may lead to unstable heteroresistance (Andersson et al., 2019; Nicoloff et al., 2019). Other mechanisms of heteroresistance have been reported for which no information regarding stability is available. Studies have suggested that low expression of the OprD porin, marginal overexpression of some efflux pump encoding genes, and intrinsic β-lactamases, may contribute to imipenem-heteroresistant *P. aeruginosa* (Ikonomidis et al., 2008; Mei et al., 2015; Xu et al., 2020). An insertion sequence was found in the promoter region of a class C β-lactamase gene in imipenem-heteroresistant multi-drug resistant *A. baumannii* strains, and was associated to overexpression of such β-lactamase. In that case, the use of carbapenem seemed to be the only risk factor identified for the emergence of carbapenem-heteroresistance (Lee et al., 2011). Also, *bla*_OXA-58-like_, *bla*_OXA-51-like_, IS*Aba2* and IS*Aba3* have been associated with *A. baumannii* heterogeneous resistance to carbapenems (Fernández Cuenca et al., 2012). In the case of *K. pneumoniae*, heteroresistant colonies were shown to have significantly elevated expression of the *bla*_KPC_ gene compared with the native populations but did not retain heteroresistance when subcultured in drug-free media (Pournaras et al., 2010); more recently, resistant and susceptible *K. pneumoniae* subpopulations have been shown to be genetically identical by genome sequencing (Sancak et al., 2022). In our strain collection, we could not establish associations between imipenem heteroresistance and overexpression of efflux pump encoding genes, β-lactamases, or imipenem use. Together, our observations suggest that imipenemen heteroresistance may be a frequent but also complex and likely multifactorial phenotype. These observations align with the notion that high prevalence of heteroresistance with the potential for treatment failure highlights the limitations of MIC as the sole criterion for susceptibility determination (Nicoloff et al., 2019). Finally, we advocate that the existing heteroresistance high rate is worth our attention, and support the need for facile and rapid protocols to identify heteroresistance in pathogens.

## 4 Materials and methods

### 4.1 Bacterial isolates and growth conditions

We used a previously sequenced set of NTHi isolates serially collected from COPD patients (Moleres et al., 2018). Thirteen patient series with four or more longitudinally sampled NTHi isolates were included. This collection consists of 90 isolates collected over a period of 1 to 9 years, belonging to 40 clonal types (CTs) (Table 1 and Figure 3). *H. influenzae* RdKW20 (Fleischmann et al., 1995) was used as an imipenem susceptible reference strain. *H. influenzae* strains were grown at 37°C, 5% CO_2_ on PolyViteX agar (PVX agar, Biomérieux, ref. 43101), Mueller-Hinton fastidious (MH-F) agar medium (MH-F, Biomérieux, ref. 43901), or on *Haemophilus* Test Medium agar (HTM, Oxoid, ref. CM0898) supplemented with 10 μg/mL hemin and 10 μg/mL nicotinamide adenine dinucleotide (NAD), referred to as sHTM agar. NTHi liquid cultures were grown at 37°C, 5% CO_2_ in brain-heart infusion (BHI, Condalab, ref. 1400.10) supplemented with 10 μg/mL hemin and 10 μg/mL NAD, referred to as sBHI.

### 4.2 Antimicrobial susceptibility testing

Imipenem susceptibility was assessed by disc diffusion (10 mg discs, Bio-Rad, ref. 66568) and Etest (Biomérieux, ref. 412374) on MH-F agar plates following the recommended clinical breakpoints of the European Committee on Antimicrobial Susceptibility Testing (EUCAST) guidelines (European committee on antimicrobial susceptibility, 2023): imipenem resistance (MIC >2 μg/mL; growth inhibition diameter < 20 mm) (Figure 1 step 1). In all cases, assays were performed in three independent occasions, using different plate batches and imipenem Etests and discs. Results were independently read by the first and last authors. The growth of colonies inside the growth inhibition halo in the presence of imipenem disc or Etest was considered as an indicator of heteroresistance. When colonies were found inside the growth inhibition halo, such colonies were collected and used as starting point to repeat the disc diffusion assay as described above (Figure 1 step 2). The β-lactamase activity was determined by the chromogenic cephalosporin test using nitrocefin as a substrate and following the manufacturer’s directions (Becton Dickinson).

### 4.3 Population analysis profiling

Imipenem PAP (Figure 1 step 3) was determined by following previously described procedures (Cerquetti et al., 2007; Cherkaoui et al., 2017, 2018a,b). Briefly, *H. influenzae* strains were grown on PVX agar for 16 h. A suspension of bacteria was then generated in 30 mL PBS and adjusted to OD_600_ = 1. Suspensions were centrifuged at 4,000 r.p.m. for 10 min, pellets were resuspended in 3 mL PBS rendering the starting inoculum dose, ~10^9^ CFU/mL, and serially diluted up to 10^−7^. The microdilution plate count was performed by plating 10 μL droplets of each dilution onto a set of sHTM agar plates containing increasing concentrations of imipenem (concentration range, 0.25–64 μg/mL) and onto antibiotic-free sHTM agar. After incubation at 37°C with 5% CO_2_ for 48 h, bacterial colonies were counted. Assays were carried out in triplicate and at least two independent occasions (n ≥ 6).

### 4.4 Tolerance disc test

The two-steps TD-test is a modification of the standard disc-diffusion assay that enables the detection of tolerant and persistent bacteria by promoting the growth of the surviving bacteria in the inhibition zone, once the antibiotic has diffused away (Gefen et al., 2017). This test, originally described for *Escherichia coli*, was adapted to *H. influenzae* in terms of growth media requirements (Figure 1 steps 1 and 4). Briefly, a standard disc diffusion assay, using 10 mg imipenem discs on MH-F agar, was followed by an antibiotic disc replacement with a 2 mg glucose-containing disc after 24 h. Then, plates were incubated at 37°C with 5% CO_2_ for 24 h. For preparation of glucose discs, 6 mm diameter filter paper discs (Whatman, ref. WHA2017006) were moistened with 5 μL of a 40% sterile glucose solution. Tolerance was assessed by the appearance of colonies within the growth inhibition zone. Controls were run in parallel by maintaining the antibiotic disc for 48 h. The growth of colonies inside the growth inhibition halo, after replacing the antibiotic disc with a glucose disc, was considered as an indicator of tolerance.

### 4.5 PBP3, AcrA, AcrB, and AcrR allelic identities for *Haemophilus influenzae* strain collection

Analysis of allelic variation of the *ftsI*, *acrA, acrB* and *acrR* genes was performed using Geneious Prime (Biomatters). A local database containing the 90 whole genome sequences was created and local blast tool was employed, with the genome of *H. influenzae* RdKW20 (NC_000907) as a reference. Subsequently, the nucleotide alignments were translated to detect amino acid changes.

### 4.6 Comparative pangenome and single nucleotide polymorphism variation analysis

The pangenomic analysis was performed to detect differences in presence or absence of genes when comparing susceptible and heteroresistant strains. Imipenem-resistant strains were excluded. A gene presence and absence matrix was created with Roary (Page et al., 2015), with a minimum percentage of identity of 70% for BLASTp, and the analysis of potential genes associated to heteroresistance was done using Scoary (github.com/AdmiralenOla/Scoary). An intra-CT SNPs analysis was performed in those CT presenting differences in terms of heteroresistance phenotype. A whole genome alignment for each CT was created by Snippy v4.4.0 (github.com/tseemann/snippy) using P595-8370 (GCA_003425765) as reference for CT14; P621-7028 (GCA_003425445) for CT48; P604-7629 (GCA_003415395) for CT72; and P625-8065 (GCA_003415195) for CT95.

### 4.7 RNA extraction, purification, and further processing

NTHi was grown for 12 h on PVX agar. Two to five colonies were then inoculated into 10 mL sBHI, grown for 12 h at 90 r.p.m., diluted into 20 mL fresh sBHI to OD_600_ = 0.05, and grown to OD_600_ = 0.6 at 200 r.p.m. In all cases, 7 mL were recovered from each culture, pelleted (4,000 r.p.m., 4 min), flash frozen, and stored at-80°C. Total RNA was isolated using TRIzol reagent (Invitrogen). Purified RNA was quantified on a Nanodrop One^C^ (Thermofisher Scientific), and total RNA integrity determined using RNA 6000 Nano LabChips (Agilent 2,100 Bioanalyzer). Reverse transcription was performed using 1 μg RNA by PrimerScript RT Reagent kit (Takara). cDNA diluted 1:10 was used as template in a 20 μL reaction mixture containing 1X SYBR Premix Ex Taq II (Tli RNaseH Plus) (Takara), and specific primers pairs for *acrA* (F1-qPCR-acrA/24905′-GATCGTCAAGGTGTTTATGCTCA;R1-qPCR-acrA/2491, 5′-CCATTACCAATACCTTGCTGACC), designed with Primer3 software. Fluorescence was analyzed with AriaMx Real-Time PCR System (Agilent Technologies). The comparative threshold cycle (Ct) method was used to obtain relative quantities of mRNA that were normalized using bacteria *gyrA* gene as endogenous control (*gyrA*-F1/1078, 5′-ATATGTTGGTTGATGGGCAAGG; *gyrA*-R1/1079, 5-GGCGAGAAATTGACGGTTTCT). Bacterial cultures were grown at least three times, and all samples were processed with triplicates (n ≥ 3).

### 4.8 Statistical analyses

In all cases, *p* < 0.05 value was considered statistically significant. Analyses were performed using Prism software, version 7 for Mac (GraphPad Software) statistical package and are detailed in the Figure Legends when needed.

## Data availability statement

The original contributions presented in the study are included in the article/supplementary material, further inquiries can be directed to the corresponding author.

## Author contributions

CG-C: Formal analysis, Methodology, Validation, Writing – original draft, Writing – review & editing. AG-D: Data curation, Investigation, Methodology, Writing – review & editing. OI-E: Investigation, Methodology, Writing – review & editing. BR-A: Data curation, Investigation, Methodology, Writing – review & editing. IE-G: Investigation, Methodology, Writing – review & editing. AF-C: Conceptualization, Formal analysis, Methodology, Writing – review & editing. ML-D: Investigation, Methodology, Writing – review & editing. SM: Conceptualization, Formal analysis, Funding acquisition, Methodology, Writing – original draft, Writing – review & editing. JG: Conceptualization, Formal analysis, Funding acquisition, Methodology, Project administration, Supervision, Writing – original draft, Writing – review & editing.

## References

[ref1] Ahearn C. P. Gallo M. C. Murphy T. F. (2017). Insights on persistent airway infection by non-typeable *Haemophilus influenzae* in chronic obstructive pulmonary disease. Pathog. Dis. 75, 1–18. doi: 10.1093/femspd/ftx042 PMC5437125 28449098

[ref2] Andersson D. I. Nicoloff H. Hjort K. (2019). Mechanisms and clinical relevance of bacterial heteroresistance. Nat. Rev. Microbiol. 17, 479–496. doi: 10.1038/s41579-019-0218-1, PMID: 31235888

[ref3] Balaban N. Q. Helaine S. Lewis K. Ackermann M. Aldridge B. Andersson D. I.. (2019). Definitions and guidelines for research on antibiotic persistence. Nat. Rev. Microbiol. 17, 441–448. doi: 10.1038/s41579-019-0196-3, PMID: 30980069 PMC7136161

[ref4] Band V. I. Weiss D. S. (2019). Heteroresistance: a cause of unexplained antibiotic treatment failure? PLoS Pathog. 15, 1–7. doi: 10.1371/journal.ppat.1007726 PMC6553791 31170271

[ref5] Brauner A. Fridman O. Gefen O. Balaban N. Q. (2016). Distinguishing between resistance, tolerance and persistence to antibiotic treatment. Nat. Rev. Microbiol. 14, 320–330. doi: 10.1038/nrmicro.2016.34, PMID: 27080241

[ref6] Carrera-Salinas A. González-Díaz A. Ehrlich R. L. Berbel D. Tubau F. Pomares X.. (2023). Genetic adaptation and acquisition of macrolide resistance in *Haemophilus* spp. during persistent respiratory tract colonization in chronic obstructive pulmonary disease (COPD) patients receiving long-term azithromycin treatment. 11:e0386022. doi: 10.1128/spectrum.03860-22 PMC9927455 36475849

[ref7] Cerquetti M. Giufrè M. Cardines R. Mastrantonio P. (2007). First characterization of heterogeneous resistance to imipenem in invasive nontypeable *Haemophilus influenzae* isolates. Antimicrob. Agents Chemother. 51, 3155–3161. doi: 10.1128/AAC.00335-07, PMID: 17620383 PMC2043221

[ref8] Cherkaoui A. Diene S. M. Fischer A. Leo S. François P. Schrenzel J. (2018a). Transcriptional modulation of penicillin-binding protein 1b, outer membrane protein P2 and efflux pump (AcrAB-TolC) during heat stress is correlated to enhanced bactericidal action of imipenem on non-typeable *Haemophilus influenzae*. Front. Microbiol. 8, 1–12. doi: 10.3389/fmicb.2017.02676 PMC5770572 29375536

[ref9] Cherkaoui A. Diene S. M. Renzoni A. Emonet S. Renzi G. François P.. (2017). Imipenem heteroresistance in nontypeable *Haemophilus influenzae* is linked to a combination of altered PBP3, slow drug influx and direct efflux regulation. Clin. Microbiol. Infect. 23, 118.e9–118.e19. doi: 10.1016/j.cmi.2016.10.009, PMID: 27756711

[ref10] Cherkaoui A. Gaïa N. Baud D. Leo S. Fischer A. Ruppe E.. (2018b). Molecular characterization of fluoroquinolones, macrolides, and imipenem resistance in *Haemophilus influenzae*: analysis of the mutations in QRDRs and assessment of the extent of the AcrAB-TolC-mediated resistance. Eur. J. Clin. Microbiol. Infect. Dis. 37, 2201–2210. doi: 10.1007/s10096-018-3362-z, PMID: 30145620

[ref11] Dean C. R. Narayan S. Daigle D. M. Dzink-Fox J. L. Puyang X. Bracken K. R.. (2005). Role of the AcrAB-TolC efflux pump in determining susceptibility of *Haemophilus influenzae* to the novel peptide deformylase inhibitor LBM415. Antimicrob. Agents Chemother. 49, 3129–3135. doi: 10.1128/AAC.49.8.3129-3135.2005, PMID: 16048914 PMC1196275

[ref12] Dewachter L. Fauvart M. Michiels J. (2019). Bacterial heterogeneity and antibiotic survival: understanding and combatting persistence and heteroresistance. Mol. Cell 76, 255–267. doi: 10.1016/j.molcel.2019.09.028 31626749

[ref13] Duell B. L. Su Y. C. Riesbeck K. (2016). Host–pathogen interactions of nontypeable *Haemophilus influenzae*: from commensal to pathogen. FEBS Lett. 590, 3840–3853. doi: 10.1002/1873-3468.12351, PMID: 27508518

[ref14] El-Halfawy O. M. Valvano M. A. (2015). Antimicrobial heteroresistance: an emerging field in need of clarity. Clin. Microbiol. Rev. 28, 191–207. doi: 10.1128/CMR.00058-14, PMID: 25567227 PMC4284305

[ref15] European committee on antimicrobial susceptibility testing (2023). Breakpoint tables for interpretation of MICs and zone diameters. Available at: https://www.eucast.org/fileadmin/src/media/PDFs/EUCAST_files/Breakpoint_tables/v_13.1_Breakpoint_Tables.pdf

[ref16] Fernández Cuenca F. Sánchez M. D. C. G. Caballero-Moyano F. J. Vila J. Martínez-Martínez L.. (2012). Prevalence and analysis of microbiological factors associated with phenotypic heterogeneous resistance to carbapenems in *Acinetobacter baumannii*. Int. J. Antimicrob. Agents 39, 472–477. doi: 10.1016/j.ijantimicag.2012.01.015, PMID: 22445494

[ref17] Fleischmann R. D. Adams M. D. White O. Clayton R. A. Kirkness E. F. Kerlavage A. R.. (1995). Whole-genome random sequencing and assembly of *Haemophilus influenzae* Rd. Science 269, 496–512. doi: 10.1126/science.7542800 7542800

[ref18] García-Cobos S. Arroyo M. Pé Rez-Vázquez M. Aracil B. Lara N. Oteo J.. (2014). Isolates of β-lactamase-negative ampicillin-resistant *Haemophilus influenzae* causing invasive infections in Spain remain susceptible to cefotaxime and imipenem. J. Antimicrob. Chemother. 69, 111–116. doi: 10.1093/jac/dkt324 23943391

[ref19] Gefen O. Chekol B. Strahilevitz J. Balaban N. Q. (2017). TDtest: easy detection of bacterial tolerance and persistence in clinical isolates by a modified disk-diffusion assay. Sci. Rep. 7, 1–9. doi: 10.1038/srep41284 28145464 PMC5286521

[ref20] Ikonomidis A. Tsakris A. Kantzanou M. Spanakis N. Maniatis A. N. Pournaras S. (2008). Efflux system overexpression and decreased OprD contribute to the carbapenem heterogeneity in *Pseudomonas aeruginosa*. FEMS Microbiol. Lett. 279, 36–39. doi: 10.1111/j.1574-6968.2007.00997.x, PMID: 18070070

[ref21] Jalalvand F. Riesbeck K. (2018). Update on non-typeable *Haemophilus influenzae*-mediated disease and vaccine development. Expert Rev. Vaccines 17, 503–512. doi: 10.1080/14760584.2018.1484286, PMID: 29863956

[ref22] Lâm T. T. Nürnberg S. Claus H. Vogel U. (2020). Molecular epidemiology of imipenem resistance in invasive *Haemophilus influenzae* infections in Germany in 2016. J. Antimicrob. Chemother. 75, 2076–2086. doi: 10.1093/jac/dkaa159 32449913

[ref23] Lee H. Y. Chen C. L. Wang S. B. Su L. H. Chen S. H. Liu S. Y.. (2011). Imipenem heteroresistance induced by imipenem in multidrug-resistant *Acinetobacter baumannii*: mechanism and clinical implications. Int. J. Antimicrob. Agents 37, 302–308. doi: 10.1016/j.ijantimicag.2010.12.015, PMID: 21353490

[ref24] Mei S. Gao Y. Zhu C. Dong C. Chen Y. (2015). Research of the heteroresistance of *Pseudomonas aeruginosa* to imipenem. Int. J. Clin. Exp. Med. 8, 6129–6132. 26131216 PMC4483920

[ref25] Moleres J. Fernández-Calvet A. Ehrlich R. L. Martí S. Pérez-Regidor L. Euba B.. (2018). Antagonistic pleiotropy in the bifunctional surface protein FadL (OmpP1) during adaptation of *Haemophilus influenzae* to chronic lung infection associated with chronic obstructive pulmonary disease. MBio 9, 1–23. doi: 10.1128/mBio.01176-18 PMC6156194 30254117

[ref26] Murray C. J. Ikuta K. S. Sharara F. Swetschinski L. Robles Aguilar G. Gray A.. (2022). Global burden of bacterial antimicrobial resistance in 2019: a systematic analysis. Lancet 399, 629–655. doi: 10.1016/S0140-6736(21)02724-0, PMID: 35065702 PMC8841637

[ref27] Nicoloff H. Hjort K. Levin B. R. Andersson D. I. (2019). The high prevalence of antibiotic heteroresistance in pathogenic bacteria is mainly caused by gene amplification. Nat. Microbiol. 4, 504–514. doi: 10.1038/s41564-018-0342-0, PMID: 30742072

[ref28] Page A. J. Cummins C. A. Hunt M. Wong V. K. Reuter S. Holden M. T. G.. (2015). Roary: rapid large-scale prokaryote pan genome analysis. Bioinformatics 31, 3691–3693. doi: 10.1093/bioinformatics/btv421, PMID: 26198102 PMC4817141

[ref29] Parr T. R. Bryan L. E. (1984). Mechanism of resistance of an ampicillin-resistant, β-lactamase-negative clinical isolate of *Haemophilus influenzae* type b to β-lactam antibiotics. Antimicrob. Agents Chemother. 25, 747–753. doi: 10.1128/AAC.25.6.747, PMID: 6611136 PMC185634

[ref30] Potts C. C. Rodriguez-Rivera L. D. Retchless A. C. Buono S. A. Chen A. T. Marjuki H.. (2022). Antimicrobial susceptibility survey of invasive *Haemophilus influenzae* in the United States in 2016. Microbiol. Spectr. 10:e0257921. doi: 10.1128/spectrum.02579-21, PMID: 35536039 PMC9241922

[ref31] Pournaras S. Kristo I. Vrioni G. Ikonomidis A. Poulou A. Petropoulou D.. (2010). Characteristics of meropenem heteroresistance in *Klebsiella pneumoniae* carbapenemase (KPC)-producing clinical isolates of *K. pneumoniae*. J. Clin. Microbiol. 48, 2601–2604. doi: 10.1128/JCM.02134-09, PMID: 20504985 PMC2897536

[ref32] Safari D. Wahyono D. J. Tafroji W. Darmawan A. B. Winarti Y. Kusdaryanto W. D.. (2022). Serotype distribution and antimicrobial resistance profile of *Haemophilus influenzae* isolated from school children with acute otitis media. Int J Microbiol 2022, 1–5. doi: 10.1155/2022/5391291 PMC9152372 35655653

[ref33] Sancak B. Arı O. Durmaz R. (2022). Whole-genome sequence analysis of carbapenem-heteroresistant *Klebsiella pneumoniae* and *Escherichia coli* isolates. Curr. Microbiol. 79, 1–9. doi: 10.1007/s00284-022-03087-x 36329236

[ref34] Stojowska-swędrzyńska K. Łupkowska A. Kuczyńska-wiśnik D. Laskowska E. (2022). Antibiotic heteroresistance in *Klebsiella pneumoniae*. 23:449. doi: 10.3390/ijms23010449 PMC8745652 35008891

[ref35] Su Y. C. Jalalvand F. Thegerström J. Riesbeck K. (2018). The interplay between immune response and bacterial infection in COPD: focus upon non-typeable *Haemophilus influenzae*. Front. Immunol. 9, 1–26. 30455693 10.3389/fimmu.2018.02530 PMC6230626

[ref36] Xu Y. Zheng X. Zeng W. Chen T. Liao W. Qian J.. (2020). Mechanisms of heteroresistance and resistance to imipenem in *Pseudomonas aeruginosa*. Infect. Drug Resist. 13, 1419–1428. doi: 10.2147/IDR.S249475, PMID: 32523360 PMC7234976

