# Supplementary material for: Imipenem heteroresistance but not tolerance in Haemophilus influenzae during chronic lung infection associated with chronic obstructive pulmonary disease
Source: Front Microbiol. 2023 Dec 20;14:1253623. doi: 10.3389/fmicb.2023.1253623 (PMC10765533; doi:10.3389/fmicb.2023.1253623)
Supplement: Supplementary file 1 [file Data_Sheet_1.pdf]

Supplemental Material

Imipenem heteroresistance but not tolerance in *Haemophilus influenzae* during chronic lung infection associated with chronic obstructive pulmonary disease

Celia Gil-Campillo[1,2,3], Aida González-Díaz[2,4], Beatriz Rapún-Araiz[1,3], Oihane Iriarte-Elizaintzin[1], Iris Elizalde-Gutiérrez[1], Ariadna Fernández-Calvet[1], María Lázaro-Díez[1], Sara Martí[2,4], Junkal Garmendia[1,2,3#]

[1]Instituto de Agrobiotecnología, Consejo Superior de Investigaciones Científicas (IdAB-CSIC)-Gobierno de Navarra, Mutilva, Spain; [2]Centro de Investigación Biomédica en Red de Enfermedades Respiratorias (CIBERES), Madrid, Spain; [3]Conexion Nanomedicina CSIC (NanomedCSIC); [4]Microbiology Department, Hospital Universitari Bellvitge, IDIBELL-UB, L´Hospitalet de Llobregat, Spain

Content:

Table S1. AcrB truncated variants identified in the *H. influenzae* strain collection under study.

Table S2. AcrR truncated variants identified in the *H. influenzae* strain collection under study.

Table S3. Summary of FtsI, ArcA, ArcB and ArcR variant distribution across *H. influenzae* strain collection.

Table S1. AcrB truncated variants identified in the *H. influenzae* strain collection under study. "." indicates no amino acid substitution; * indicates early stop codon.

Strain	Amino acid substitution for:																																																
Rd KW20	M 1	K 2	F 3	T 4	D 5	I 6	F 7	I 8	R 9	R 10	P 11	V 12	L 13	A 14	V 15	S 16	I 17	S 18	L 19	L 20	M 21	I 22	I 23	L 24	G 25	L 26	Q 27	A 28	I 29	S 30	K 31	L 32	A 33	V 34	R 35	E 36	Y 37	P 38	K 39	M 40	T 41	T 42	T 43	v 44	I 45	T 46	V 47	S 48	T 49
P615	.	.	.	.	.	.	.	.	.	.	.	.	.	.	.	.	.	.	.	.	.	.	.	.	.	.	.	.	.	.	.	.	.	.	.	.	.	.	.	.	.	.	.	.	.	.	.	.	.
P616	.	.	.	.	.	.	.	.	.	.	.	.	.	.	.	.	.	.	.	.	.	.	.	.	.	.	.	.	.	.	.	.	.	.	.	.	.	.	.	.	.	.	.	.	.	.	.	.	.
P600	.	.	.	.	.	.	.	.	.	.	.	.	.	.	.	.	.	.	.	.	.	.	.	.	.	.	.	.	.	.	.	.	.	.	.	.	.	.	.	.	.	.	.	.	.	.	.	.	.
P601	.	.	.	.	.	.	.	.	.	.	.	.	.	.	.	.	.	.	.	.	.	.	.	.	.	.	.	.	.	.	.	.	.	.	.	.	.	.	.	.	.	.	.	.	.	.	.	.	.
P602	.	.	.	.	.	.	.	.	.	.	.	.	.	.	.	.	.	.	.	.	.	.	.	.	.	.	.	.	.	.	.	.	.	.	.	.	.	.	.	.	.	.	.	.	.	.	.	.	.

Rd KW20	Y 51	P 52	G 53	A 54	D 55	A 56	N 57	L 58	I 59	Q 60	A 61	F 62	V 63	T 64	S 65	K 66	L 67	E 68	E 69	S 70	I 71	A 72	Q 73	A 74	D 75	N 76	I 77	D 78	Y 79	M 80	S 81	S 82	T 83	S 84	A 85	P 86
P615	.	.	.	.	.	.	.	.	.	.	.	.	.	.	.	.	.	.	.	.	.	.	.	.	.	.	.	.	.	I	L	Y	V	F	D	*
P616	.	.	.	.	.	.	.	.	.	.	.	.	.	.	.	.	.	.	.	.	.	.	.	.	.	.	.	.	.	I	L	Y	V	F	D	*
P600	.	.	.	.	.	.	.	.	.	.	.	.	.	.	.	.	.	.	.	.	.	.	.	.	.	.	.	.	.	I	L	Y	V	F	D	*
P601	.	.	.	.	.	.	.	.	.	.	.	.	.	.	.	.	.	.	.	.	.	.	.	.	.	.	.	.	.	I	L	Y	V	F	D	*
P602	.	.	.	.	.	.	.	.	.	.	.	.	.	.	.	.	.	.	.	.	.	.	.	.	.	.	.	.	.	I	L	Y	V	F	D	*

Table S2. AcrR truncated variants identified in the *H. influenzae* strain collection under study. "." indicates no amino acid substitution; * indicates early stop codon.

Strain	Amino acid substitution for:																																																	
**Rd**	M	R	Q	A	K	T	D	L	A	E	Q	I	F	S	A	T	D	R	L	M	A	R	E	G	L	N	Q	L	S	M	L	K	L	A	K	E	A	N	V	A	A	G	T	I	Y	L	Y	F	K	N	K
**KW20**	1	2	3	4	5	6	7	8	9	10	11	12	13	14	15	16	17	18	19	20	21	22	23	24	25	26	27	28	29	30	31	32	33	34	35	36	37	38	39	40	41	42	43	44	45	46	47	48	49	50	51
**P612**	.	.	.	.	.	.	.	.	.	.	.	.	.	.	.	.	.	.	.	.	.	K	.	.	.	.	.	.	.	.	H	.	.	.	.	.	.	.	.	.	.	.	.	.	.	.	.	.	.	.	.
**P615**	.	.	.	.	.	.	.	.	.	.	.	.	.	.	.	.	.	.	.	.	.	K	.	.	.	.	.	.	.	.	H	.	.	.	.	.	.	.	.	.	.	.	.	.	.	.	.	.	.	.	.
**P616**	.	.	.	.	.	.	.	.	.	.	.	.	.	.	.	.	.	.	.	.	.	K	.	.	.	.	.	.	.	.	H	.	.	.	.	.	.	.	.	.	.	.	.	.	.	.	.	.	.	.	.
**P620**	.	.	.	.	.	.	.	.	.	.	.	.	.	.	.	.	.	.	.	.	.	K	.	.	.	.	.	.	.	.	H	.	.	.	.	.	.	.	.	.	.	.	.	.	.	.	.	.	.	.	.
**P621**	.	.	.	.	.	.	.	.	.	.	.	.	.	.	.	.	.	.	.	.	.	K	.	.	.	.	.	.	.	.	H	.	.	.	.	.	.	.	.	.	.	.	.	.	.	.	.	.	.	.	.
**P623**	.	.	.	.	.	.	.	.	.	.	.	.	.	.	.	.	.	.	.	.	.	K	.	.	.	.	.	.	.	.	H	.	.	.	.	.	.	.	.	.	.	.	.	.	.	.	.	.	.	.	.
**P624**	.	.	.	.	.	.	.	.	.	.	.	.	.	.	.	.	.	.	.	.	.	K	.	.	.	.	.	.	.	.	H	.	.	.	.	.	.	.	.	.	.	.	.	.	.	.	.	.	.	.	.
**P629**	.	.	.	.	.	.	.	.	.	.	.	.	.	.	.	.	.	.	.	.	.	K	.	.	.	.	.	.	.	.	H	.	.	.	.	.	.	.	.	.	.	.	.	.	.	.	.	.	.	.	.
**P600**	.	.	.	.	.	.	.	.	.	.	.	.	.	.	.	.	.	.	.	.	.	K	.	.	.	.	.	.	.	.	H	.	.	.	.	.	.	.	.	.	.	.	.	.	.	.	.	.	.	.	.
**P601**	.	.	.	.	.	.	.	.	.	.	.	.	.	.	.	.	.	.	.	.	.	K	.	.	.	.	.	.	.	.	H	.	.	.	.	.	.	.	.	.	.	.	.	.	.	.	.	.	.	.	.
**P645**	.	.	.	.	.	.	.	.	.	.	.	.	.	.	.	.	.	.	.	.	.	.	.	.	.	.	.	.	.	.	H	.	.	.	.	.	.	.	.	.	.	.	.	.	.	.	.	.	.	.	.
**P602**	.	.	.	.	.	.	.	.	.	.	.	.	.	.	.	.	.	.	.	.	.	K	.	.	.	.	.	.	.	.	H	.	.	.	.	.	.	.	.	.	.	.	.	.	.	.	.	.	.	.	.
**P613**	.	.	.	.	.	.	.	.	.	.	.	.	.	.	.	.	.	.	.	.	.	K	.	.	.	.	.	.	.	.	H	.	.	.	.	.	.	.	.	.	.	.	.	.	.	.	.	.	.	.	.
**P614**	.	.	.	.	.	.	.	.	.	.	.	.	.	.	.	.	.	.	.	.	.	K	.	.	.	.	.	.	.	.	H	.	.	.	.	.	.	.	.	.	.	.	.	.	.	.	.	.	.	.	.
**P618**	.	.	.	.	.	.	.	.	.	.	.	.	.	.	.	.	.	.	.	.	.	K	.	.	.	.	.	.	.	.	H	.	.	.	.	.	.	.	.	.	.	.	.	.	.	.	.	.	.	.	.
**P622**	.	.	.	.	.	.	.	.	.	.	.	.	.	.	.	.	.	.	.	.	.	K	.	.	.	.	.	.	.	.	H	.	.	.	.	.	.	.	.	.	.	.	.	.	.	.	.	.	.	.	.
**P632**	.	.	.	.	.	.	.	.	.	.	.	.	.	.	.	.	.	.	.	.	.	K	.	.	.	.	.	.	.	.	H	.	.	.	.	.	.	.	.	.	.	.	.	.	.	.	.	.	.	.	.
**P633**	.	.	.	.	.	.	.	.	.	.	.	.	.	.	.	.	.	.	.	.	.	K	.	.	.	.	.	.	.	.	H	.	.	.	.	.	.	.	.	.	.	.	.	.	.	.	.	.	.	.	.
**P657**	.	.	.	.	.	.	.	.	.	.	.	.	.	.	.	.	.	.	.	.	.	.	.	.	.	.	.	.	.	.	H	.	.	.	.	.	.	.	.	E	K	H	L	V	F	.	T	R	.	S	H
**P658**	.	.	.	.	.	.	.	.	.	.	.	.	.	.	.	.	.	.	.	.	.	.	.	.	.	.	.	.	.	.	H	.	.	.	.	.	.	.	.	E	K	H	L	V	F	.	T	R	.	S	H
**P660**	.	.	.	.	.	.	.	.	.	.	.	.	.	.	.	.	.	.	.	.	.	.	.	.	.	.	.	.	.	.	H	.	.	.	.	.	.	.	.	E	K	H	L	V	F	.	T	R	.	S	H
**P591**	.	*																																																	
**P851**	.	.	*																																																
**P592**	.	.	*																																																

Strain																																																
**Rd**	D	E	L	L	E	Q	F	A	H	R	V	F	S	M	F	M	A	T	L	E	K	D	F	D	E	T	K	P	F	F	E	Q	Y	R	Q	M	W	K	N	I	W	Y	F	L	Q	E	N	P
**KW20**	52	53	54	55	56	57	58	59	60	61	62	63	64	65	66	67	68	69	70	71	72	73	74	75	76	77	78	79	80	81	82	83	84	85	86	87	88	89	90	91	92	93	94	95	96	97	98	99
**P612**	.	.	.	.	.	.	.	.	.	.	.	.	.	.	.	.	.	.	.	.	.	.	.	.	.	.	.	.	.	.	.	.	.	I	D	K	C	G	K	T	F	G	I	S	Y	K	K	I
**P615**	.	.	.	.	.	.	.	.	.	.	.	.	.	.	.	.	.	.	.	.	.	.	.	.	.	.	.	.	.	.	.	.	.	I	D	K	C	G	K	T	F	G	I	S	Y	K	K	I
**P616**	.	.	.	.	.	.	.	.	.	.	.	.	.	.	.	.	.	.	.	.	.	.	.	.	.	.	.	.	.	.	.	.	.	I	D	K	C	G	K	T	F	G	I	S	Y	K	K	I
**P620**	.	.	.	.	.	.	.	.	.	.	.	.	.	.	.	.	.	.	.	.	.	.	.	.	.	.	.	.	.	.	.	.	.	I	D	K	C	G	K	T	F	G	I	S	Y	K	K	I
**P621**	.	.	.	.	.	.	.	.	.	.	.	.	.	.	.	.	.	.	.	.	.	.	.	.	.	.	.	.	.	.	.	.	.	I	D	K	C	G	K	T	F	G	I	S	Y	K	K	I
**P623**	.	.	.	.	.	.	.	.	.	.	.	.	.	.	.	.	.	.	.	.	.	.	.	.	.	.	.	.	.	.	.	.	.	I	D	K	C	G	K	T	F	G	I	S	Y	K	K	I
**P624**	.	.	.	.	.	.	.	.	.	.	.	.	.	.	.	.	.	.	.	.	.	.	.	.	.	.	.	.	.	.	.	.	.	I	D	K	C	G	K	T	F	G	I	S	Y	K	K	I
**P629**	.	.	.	.	.	.	.	.	.	.	.	.	.	.	.	.	.	.	.	.	.	.	.	.	.	.	.	.	.	.	.	.	.	I	D	K	C	G	K	T	F	G	I	S	Y	K	K	I
**P600**	.	.	.	.	.	.	.	.	.	.	.	.	.	.	.	.	.	.	.	.	.	.	.	.	.	.	.	.	.	.	.	.	.	I	D	K	C	G	K	T	F	G	I	S	Y	K	K	I
**P601**	.	.	.	.	.	.	.	.	.	.	.	.	.	.	.	.	.	.	.	.	.	.	.	.	.	.	.	.	.	.	.	.	.	I	D	K	C	G	K	T	F	G	I	S	Y	K	K	I
**P645**	.	.	.	.	.	.	.	.	.	.	.	.	.	.	.	.	.	.	.	.	.	.	.	.	.	.	.	.	.	.	.	.	.	.	.	.	.	.	.	.	.	.	.	.	*			
**P602**	.	.	.	.	.	.	.	.	.	.	.	.	.	.	.	.	.	.	.	.	.	.	.	.	.	.	.	.	.	.	.	.	.	I	D	K	C	G	K	T	F	G	I	S	Y	K	K	I
**P613**	.	.	.	.	.	.	.	.	.	.	.	.	.	.	.	.	.	.	.	.	.	.	.	.	.	.	.	.	.	.	.	.	.	I	D	K	C	G	K	T	F	G	I	S	Y	K	K	I
**P614**	.	.	.	.	.	.	.	.	.	.	.	.	.	.	.	.	.	.	.	.	.	.	.	.	.	.	.	.	.	.	.	.	.	I	D	K	C	G	K	T	F	G	I	S	Y	K	K	I
**P618**	.	.	.	.	.	.	.	.	.	.	.	.	.	.	.	.	.	.	.	.	.	.	.	.	.	.	.	.	.	.	.	.	.	I	D	K	C	G	K	T	F	G	I	S	Y	K	K	I
**P622**	.	.	.	.	.	.	.	.	.	.	.	.	.	.	.	.	.	.	.	.	.	.	.	.	.	.	.	.	.	.	.	.	.	I	D	K	C	G	K	T	F	G	I	S	Y	K	K	I
**P632**	.	.	.	.	.	.	.	.	.	.	.	.	.	.	.	.	.	.	.	.	.	.	.	.	.	.	.	.	.	.	.	.	.	I	D	K	C	G	K	T	F	G	I	S	Y	K	K	I
**P633**	.	.	.	.	.	.	.	.	.	.	.	.	.	.	.	.	.	.	.	.	.	.	.	.	.	.	.	.	.	.	.	.	.	I	D	K	C	G	K	T	F	G	I	S	Y	K	K	I
**P657**	Y	L	I	Q	F	K	A	I	*																																							
**P658**	Y	L	I	Q	F	K	A	I	*																																							
**P660**	Y	L	I	Q	F	K	A	I	*																																							

Table S2 (*continued*).

Strain	Amino acid substitution for:						
**Rd KW20**	T 100	I 101	L 102	S 103	N 104	L 105	K 106
**P612**	P	L	F	Y	P	I	*
**P615**	P	L	F	Y	P	I	*
**P616**	P	L	F	Y	P	I	*
**P620**	P	L	F	Y	P	I	*
**P621**	P	L	F	Y	P	I	*
**P623**	P	L	F	Y	P	I	*
**P624**	P	L	F	Y	P	I	*
**P629**	P	L	F	Y	P	I	*
**P600**	P	L	F	Y	P	I	*
**P601**	P	L	F	Y	P	I	*
**P602**	P	L	F	Y	P	I	*
**P613**	P	L	F	Y	P	I	*
**P614**	P	L	F	Y	P	I	*
**P618**	P	L	F	Y	P	I	*
**P622**	P	L	F	Y	P	I	*
**P632**	P	L	F	Y	P	I	*
**P633**	P	L	F	Y	P	I	*

Table S3. Summary of FtsI, ArcA, ArcB and ArcR variant distribution across *H. influenzae* strain collection. Information summarized in four groups: (i) four-gene variant combinations present in susceptible, heteroresistant and resistant strains; (ii) four-gene variant combinations present in susceptible strains; (iii) four-gene variant combinations present in heteroresistant strains; (iv) four-gene variant combinations present in resistant strains.

Clonal Type (CT)	Patient	Phenotype	Allelic variant FtsI	Allelic variant AcrA	Allelic variant AcrB	Allelic variant AcrR
14	2, 10	S, H1	12	10	11	8
17, 47	8, 12	S, H1	13	11	12	4
48	4, 6	S, H1, H2	8	6	Truncated	Truncated
48	6, 7, 8	S, H2	8	6	8	Truncated
27, 72	1, 5	S, H1	14	4	6	4
95	7, 13	R, H2	5	3	5	3
3, 28, 106	12, 13	S	19	4	12	4
6	7	S	20	14	16	3
7	13	S	1	6	17	11
16	3	S	19	4	18	4
35	6	S	19	4	19	4
38	10	S	21	15	20	Truncated
45	3	S	22	16	21	12
52	5	S	15	17	22	6
54	10	S	17	13	23	10
59	2	S	23	18	24	13
76	12	S	23	18	25	14
100	11	S	23	19	13	Truncated
107	1	S	24	7	26	Truncated
119	5	S	25	20	27	15
137	4, 8	S	17	4	28	4
138	6	S	14	4	25	4
140	3	S	26	21	29	Truncated
145	5	S	21	11	12	4
146	1	S	27	21	30	Truncated
8, 87	6, 10	H1, H2	10	8	9	3
9	1	H1	11	9	10	7
18	10	H2	6	4	6	4
40	5	H1, H2	7	5	7	5
73	4, 10	H1, H2	9	7	5	6
83	10	H1	15	7	5	6
91	10	H1	16	12	13	8
105	11	H1	17	13	14	9
124	8	H1	18	4	12	4
135	2	H1	17	13	15	10
44	6, 9	R	2	2	2	2
44	6, 9	R	3	2	3	2
44	6, 9	R	4	2	4	2
